# FP-Zernike: An Open-source Structural Database Construction Toolkit for Fast Structure Retrieval

**DOI:** 10.1093/gpbjnl/qzae007

**Published:** 2024-01-19

**Authors:** Junhai Qi, Chenjie Feng, Yulin Shi, Jianyi Yang, Fa Zhang, Guojun Li, Renmin Han

**Affiliations:** Research Center for Mathematics and Interdisciplinary Sciences, Shandong University, Qingdao 266237, China; BioMap Research, Menlo Park, CA 94025, USA; Research Center for Mathematics and Interdisciplinary Sciences, Shandong University, Qingdao 266237, China; College of Medical Information and Engineering, Ningxia Medical University, Yinchuan 750004, China; Research Center for Mathematics and Interdisciplinary Sciences, Shandong University, Qingdao 266237, China; Research Center for Mathematics and Interdisciplinary Sciences, Shandong University, Qingdao 266237, China; Institute of Engineering Medicine, Beijing Institute of Technology, Beijing 100081, China; Research Center for Mathematics and Interdisciplinary Sciences, Shandong University, Qingdao 266237, China; Research Center for Mathematics and Interdisciplinary Sciences, Shandong University, Qingdao 266237, China

**Keywords:** Zernike descriptor, Structure alignment, PDB dataset, Open-source, Retrieval system

## Abstract

The release of AlphaFold2 has sparked a rapid expansion in protein model databases. Efficient protein structure retrieval is crucial for the analysis of structure models, while measuring the similarity between structures is the key challenge in structural retrieval. Although existing structure alignment algorithms can address this challenge, they are often time-consuming. Currently, the state-of-the-art approach involves converting protein structures into three-dimensional (3D) Zernike descriptors and assessing similarity using Euclidean distance. However, the methods for computing 3D Zernike descriptors mainly rely on structural surfaces and are predominantly web-based, thus limiting their application in studying custom datasets. To overcome this limitation, we developed FP-Zernike, a user-friendly toolkit for computing different types of Zernike descriptors based on feature points. Users simply need to enter a single line of command to calculate the Zernike descriptors of all structures in customized datasets. FP-Zernike outperforms the leading method in terms of retrieval accuracy and binary classification accuracy across diverse benchmark datasets. In addition, we showed the application of FP-Zernike in the construction of the descriptor database and the protocol used for the Protein Data Bank (PDB) dataset to facilitate the local deployment of this tool for interested readers. Our demonstration contained 590,685 structures, and at this scale, our system required only 4–9 s to complete a retrieval. The experiments confirmed that it achieved the state-of-the-art accuracy level. FP-Zernike is an open-source toolkit, with the source code and related data accessible at https://ngdc.cncb.ac.cn/biocode/tools/BT007365/releases/0.1, as well as through a webserver at http://www.structbioinfo.cn/.

## Introduction

Proteins, as the building blocks of all living systems, fold into specific three-dimensional (3D) configurations and perform corresponding biological functions. To understand the mechanism of protein action at the molecular level, it is necessary to accurately predict the 3D structure of proteins. AlphaFold2 [1] has made significant strides in protein structure prediction, achieving comparable prediction accuracy with experimental methods through a well-designed deep neural network and greatly reducing the prediction time. This breakthrough suggests that the protein model structure database will grow at an amazing speed. The latest AlphaFold database release contains over 200 million entries, highlighting the urgent need for an efficient and accurate method to measure protein structural similarity.

Structural alignment is the most direct method to measure the similarity between structures. There are two main types of structural alignment methods: coordinate-based methods and surface-based methods. Coordinate-based methods, dating back to the 1970s [2], focus on the superimposition of structures based on atomic coordinate information. In the following decades, various schemes have been proposed and improved, such as combinatorial extension (CE) [3], DALI [4], RNA-align [5], TM-align [6], and US-align [7]. Since the nature of the protein surface is crucial to the study of protein–protein (RNA, ligand) interactions, structure alignment schemes based on the protein surface have been proposed, such as gmfit [8], ZEAL [9], and iterative closest point (ICP) [10]. However, these alignment methods are often time-consuming. For example, a standard alignment software (gmfit) takes ∼ 0.71 s to complete a structural alignment and ∼ 5 days for a structure retrieval (∼ 590,000 alignments).

To enhance the efficiency of measuring the similarity between protein structures, a common scheme involves converting protein structures into feature vectors, designing a metric applied to feature vectors, and transforming the problem of measuring the similarity between structures into a measurement calculation between feature vectors. In recent years, two main approaches have been employed for representing structures as feature vectors. One way is to calculate geometric information based on atomic coordinates and convert this information into feature vectors. For example, Omokage [11] calculates the distance distribution between feature atoms and proposes the Omokage score to measure the similarity between protein structures. Similarly, in BioZernike’s [12] geometric (GEO) module, the geometric information of atoms is converted into a feature vector of 17 lengths by calculating geometric features. Another way is to convert the protein structure into a protein surface and then calculate the feature vectors based on the protein surface. For example, the farthest point sampling-enhanced triangulation-based iterative-closest-point (FTIP) [13] extracts 10–90 feature points on the protein surface through the farthest point sampling algorithm and then rapidly calculates the similarity between protein structures based on the feature points and triangulation-based iterative-closest-point for protein surface alignment (TIPSA) algorithm [14]. Based on deep learning, the Surface ID [15] method encodes the surface of the protein into a fixed-dimensional feature vector. 3D-SURFER [16–18] provides a webserver for computing 3D Zernike descriptors [19] based on protein surface information (surface-based 3DZD), and BioZernike was subsequently developed for calculating 3D Zernike descriptors based on gmconvert [20].

Zernike descriptors have been widely used not only for retrieval problems but also in other areas of structural biology, including interface (binding site) prediction [21–25], embedding of polymers into Electron Microscopy (EM) maps [26], docking problems [27], and analysis of protein surfaces [28]. Furthermore, Zernike descriptors have also shown their efficacy in image reconstruction [29] and 3D structure classification [30]. Despite their versatility, there is currently a lack of open-source, easy-to-use Zernike implementation for protein or RNA structure.

This study introduces FP-Zernike, which offers a unique capability to compute different Zernike descriptors based on different structural representations, such as surface, mesh, and atomic point cloud. FP-Zernike provides two main functions for users: (1) construction of a descriptor dataset for structural analysis using a customized structural dataset, and (2) ultra-fast retrieval (4–9 s) of structure within a large dataset (including 590,685 structures) based on a query structure. Specifically, the functions have been seamlessly integrated into a webserver (see “Code availability”) for user convenience. Comprehensive experiments demonstrated that FP-Zernike could handle structure retrieval problems efficiently, outperforming the surface-based 3DZD and a surface-based alignment tool on benchmark datasets, with a top-10 accuracy of ∼ 90% and an area under the curve (AUC) value of ∼ 96%. Furthermore, the FP-Zernike-based retrieval system (FP-System) was confirmed completely feasible for large-scale structural retrieval challenges. When provided with query structures, the system yields the top 10–150 structures and calculates the average template modelling score (TM-score) between them and the query structures. Notably, the average TM-score of the FP-System is 0.15–0.23 higher than that of the state-of-the-art retrieval system based on surface-based 3DZD.

## Method

### Benchmark datasets

The benchmark datasets were meticulously constructed based on the basic local alignment search tool [31], TM-align, and the webserver [32]. To evaluate the initial performance of FP-Zernike, we constructed two protein structure datasets and one RNA structure dataset, denoted by Protein160, Protein13, and RNA16. Each dataset exhibited different characteristics, with the details presented in Table S1. In addition, to test the binary classification ability of FP-Zernike, we obtained a set of structure pairs from the aforementioned three datasets. All the structure pairs were categorized into positive and negative samples, with the detailed information provided in Table S2. To address the excessive imbalance in the numbers of positive and negative samples, we conducted random sampling to ensure a balanced ratio of positive samples to negative samples (1:3). To test the performance of FP-System, we randomly selected 500 structures from the entire single-stranded structure database to form a dataset called Random500. All details of constructing the datasets are provided in File S1 (see “Benchmark dataset construction”).

### Reference methods for comparative analysis

We conducted a comparison of our method with 3D-SURFER and gmfit. Currently, 3D-SURFER is the state-of-the-art protein structure retrieval system (including 606,272 structure chains) based on surface-based 3DZD, and gmfit can quickly complete the structure comparison on the Gaussian mixture model (GMM), enabling users to pre-calculate the GMM database and subsequently build a retrieval system based on gmfit. Please refer to File S1 (see “Reference methods”) for details on the use of 3D-SURFER and gmfit.

### Experimental environment

All the experiments were run on an Ubuntu 20.04 system with Intel Core i9-10980XE (18 cores), 128 GB memory, and an NVIDIA RTX 3080.

### FP-Zernike

#### Overview of FP-Zernike

**Figure 1:**
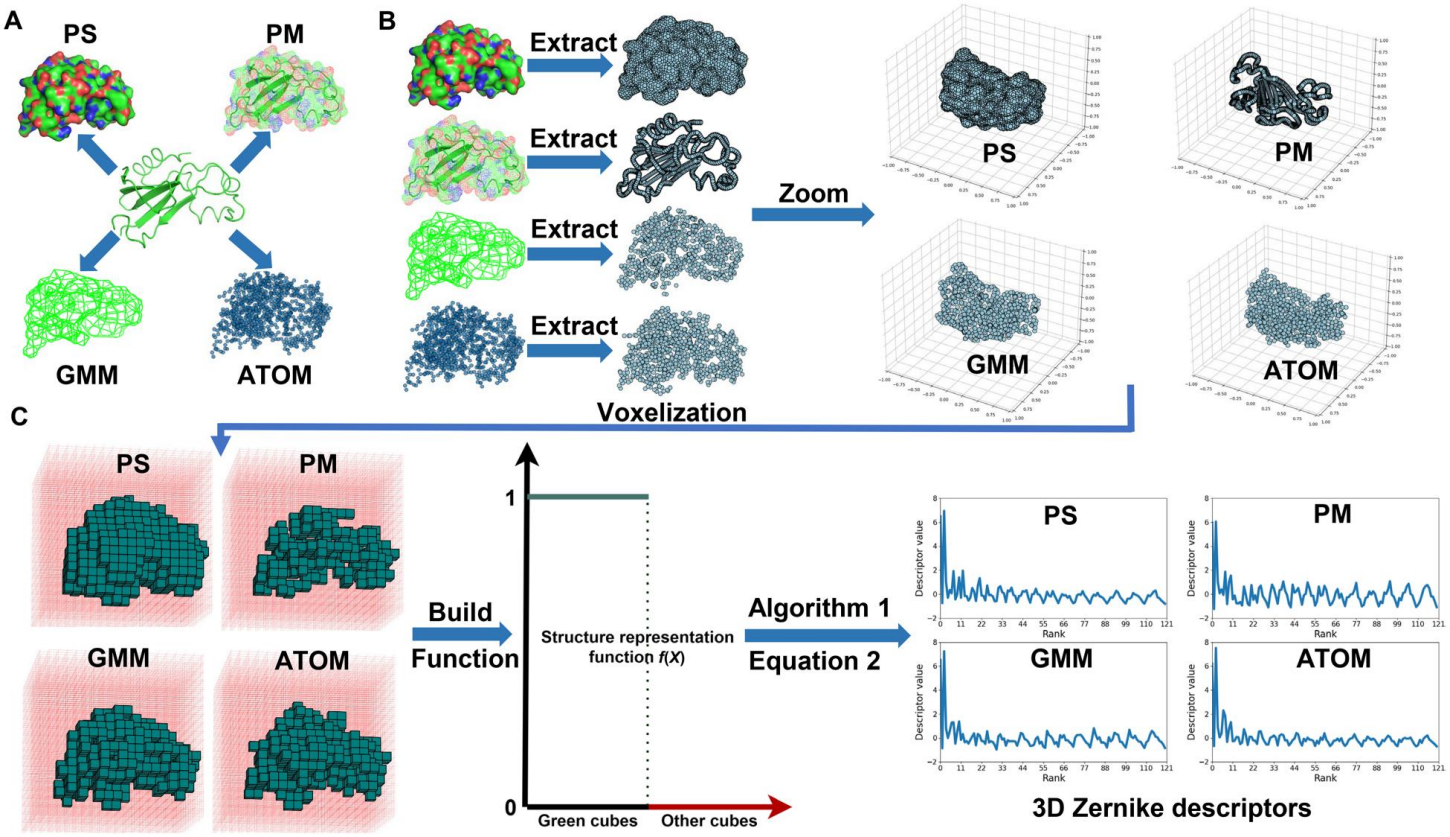
Workflow for computing 3D Zernike descriptors based on the FP-Zernike algorithm **A**. Computation of four structural representations in four modes from a given protein structure (PDB: 1brn, chain L). **B**. Extraction and scaling of feature points from each structure representation into unit spheres. **C**. Generation of the grid defined at $[-1,1{]}^{3}$ based on the feature points, with a cube symbol representing the feature points. The structure representation function f(X) is defined on the grid, taking values of 1 on the cubes representing feature points and 0 on the other cubes. Using Algorithm 1 and Equation 2, four different 3D Zernike descriptors can be computed. 3D, three-dimensional; PDB, Protein Data Bank; PS, protein surface; PM, protein mesh; GMM, Gaussian mixture model; ATOM, the atomic coordinates of the structure.

The process of computing the 3D Zernike descriptors of FP-Zernike encompassed five steps: (1) the structural representation of surfaces, meshes, and atomic point clouds was obtained; (2) feature points were extracted and scaled into the unit sphere; (3) a function was built based on the feature points to characterize the structure (Equation 1); (4) the corresponding geometric moments were calculated; and (5) specific mathematical expressions were used to compute the 3D Zernike descriptors. These five steps are briefly depicted in **Figure 1**. In addition, we improved Omokage and integrated it into the FP-Zernike to develop an offline algorithm called ReOmokage for computing the geometric features of the structures. In the context of measuring structural similarity, ReOmokage demonstrated superior performance compared with Omokage (File S1, see “Experimental details for the comparison of ReOmokage and Omokage”).

#### Representation of the structure

The core step in computing the 3D Zernike descriptors is the construction of an appropriate function f(x,y,z) to express the structure. The initial phase in constructing the function f(x,y,z) entails transforming the structure into a reasonable representation. The PyMOL (http://www.pymol.org/pymol) and gmconvert were utilized in FP-Zernike to transform protein structures into multiple forms, including calculating the surface of protein structure (PS), protein mesh (PM), and GMM to represent the structure and extracting the atomic coordinates of the structure (ATOM) to represent the structure. All structural representations are shown in Figure 1A.

#### Extraction of feature points

**Figure 2:**
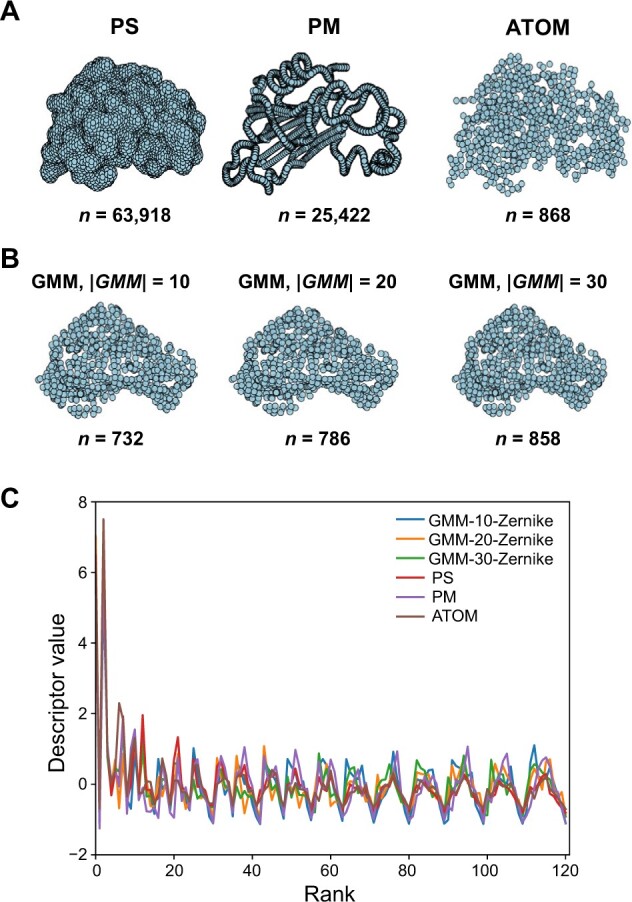
Comparison of feature point numbers and 3D Zernike descriptors in various modes of FP-Zernike **A**. Comparison of the shapes and numbers of feature points in PS, PM, and ATOM modes. *n* represents the number of feature points. **B**. Analysis of the shapes and numbers of feature points in GMM mode for different GMM parameters. *n* represents the number of feature points. **C**. Comparison of 3D Zernike descriptors in six modes. GMM, Gaussian mixture model.

To expedite the construction of the structure representation function, we extracted feature points from the structure representations, with the protein 1brn.pdb (chain L) as an example. Different schemes were employed to extract feature points for different structural representations, resulting in varying final numbers of feature points. **Figure 2** shows the feature point information and 3D Zernike descriptors of the structure of protein 1brn.pdb (chain L) under different representations. The illustration revealed that the number of feature points generated based on PS was the largest, allowing these feature points to effectively represent the shape of the structure. In ATOM and GMM modes, the number of feature points was similar, and the GMM setting had minimal effects on feature point extraction. In addition, the descriptors in different modes exhibited slight variations due to differences in feature points.

The feature points from different schemes possess different properties. For example, while a large number of feature points can be extracted based on the surface of the structure, these feature points may not effectively convey the internal information of the structure. Conversely, feature points extracted based on atomic coordinates can capture such internal information. Given the requirements of different structural biology problems, our method provides different schemes for calculating the feature points of the structure to accommodate these varied needs.

#### Structure representation function

All the feature points were embedded in the unit sphere. Let the set of feature points be denoted by S, where for all ∀(x,y,z)∈S, it satisfied $|x{|}^{2}+|y{|}^{2}+|z{|}^{2}\leq 1$. In addition, a grid (N×N×N, default N=200) was constructed on the interval $[-1,1{]}^{3}$, comprising a total of $N^{3}$ cells, and the set of cells was defined as $C=\{\mathrm{cel}l_{\left(0,0,0\right)},\mathrm{cel}l_{\left(1,0,0\right)},\ldots ,\mathrm{cel}l_{\left(N-1,N-1,N-1\right)}\}$. Equation 1 represented a mathematical expression for the structural representation function.

(1) $$\begin{array}{c} f\left(x,y,z\right)=\left\{\begin{array}{c} 1,\;if\;\left(x,y,z\right)\in \mathrm{cel}l_{\left(i,j,k\right)}\;\mathrm{and}\;\exists s\in S,\;\\ s.t.\;s\in \mathrm{cel}l_{\left(i,j,k\right)},\;0\leq i,j,k<N\\ 0,\;\mathrm{otherwise}\; \end{array}\right.\; \end{array}$$

#### FP-Zernike for computing 3D Zernike descriptors

The computation of the 3D Zernike descriptor essentially involved computing 3D Zernike moments. $\Omega_{nl}^{m}$ in Equation 2 defined the 3D Zernike moments. Here, n, l, and m were integers, l∈[0,n]; n-l was even, m∈[-l,l]; and $X\in R^{3}$. The detailed analysis for $\Omega_{nl}^{m}$ is provided in File S1 (see “Analysis of moments and 3D Zernike moments”).

Upon calculating all $\Omega_{nl}^{m}$ values, the 3D Zernike descriptor was obtained. $\chi_{\mathrm{nlm}}^{\mathrm{rst}}$ was defined in Equation 3, where k=(n-l)/2, and r,s, and t were positive integers and satisfies r+s+t≤n. The definitions of $q_{kl}^{v}$ and $c_{l}^{m}$ in Equation 3 are presented in the File S1 (see “3D Zernike descriptor”). Equations 2–4 demonstrated that $\chi_{\mathrm{nlm}}^{\mathrm{rst}}$ and f(X) were independent, indicating no dependence on the computation of $\chi_{\mathrm{nlm}}^{\mathrm{rst}}$ and f(X). Thus, $\chi_{\mathrm{nlm}}^{\mathrm{rst}}$ could be computed when order n is given. Therefore, the key to calculating the 3D Zernike descriptor was to construct f(X) and calculate $M_{\mathrm{rst}}\;$(geometric moment). Here, we defined $\Omega_{nl}=(\Omega_{nl}^{-l},\Omega_{nl}^{-l+1},\ldots ,\Omega_{nl}^{l})\;\mathrm{and}\;F_{n,l}=|\Omega_{nl}|$, and then the 3D Zernike descriptor (order=n) of f(X) was defined as the following vector $D_{f}$ in Equation 5.

(2) Ωnlm≔3π4⋅∑r+s+t≤nχnlmrst^Mrst 

(3) χnlmrst:=clm2-m∑v=0kqklv∑α=0vvα⋅∑β=0v-αv-αβ⋅∑u=0m(-1)m-u⋅muiu∑μ=0l-m2(-1)μ2-2μlμ⋅l-μm+μ∑v=0μμv

(4) Mrst≔∫X<1fXxrysztdX,X=x,y,z 

(5) Df=Fn,l0,Fn,l1,…,Fn,lt 

Based on previous work and the definition of an integral, the discrete form of Equation 4 is derived, resulting in Equation 6. The default value of N was set to 200 in our algorithm.

(6) Mrst:=∑i=0N−1 ∑j=0N-1 ∑k=0N−1 xi+1r+1-xir+1r+1⋅yj+1s+1-yjs+1s+1⋅zk+1t+1-xkt+1t+1⋅fxi,yj,zk 

Notably, the structure representation function f(X) in Equation 1 was defined on the interval $[-1,1{]}^{3}$. For ∀s∈S, it held that |s|<1. Let cube={(x,y,z)| |x|, |y|, |z|≤1} and $\mathrm{unitBall}=\{(x,y,z)|\;x^{2}+y^{2}+z^{2}\leq 1\}$, the following formula held:

(7) ∫cubefXxrysztdX=∫unitBallfXxrysztdX+∫cube∖unitBallfXxrysztdX=∫unitBallfXxrysztdX=∫X<1fXxrysztdX=Mrst 

Therefore, the integral of f(X) on cube was equivalent to the integral of f(X) on unitBall.

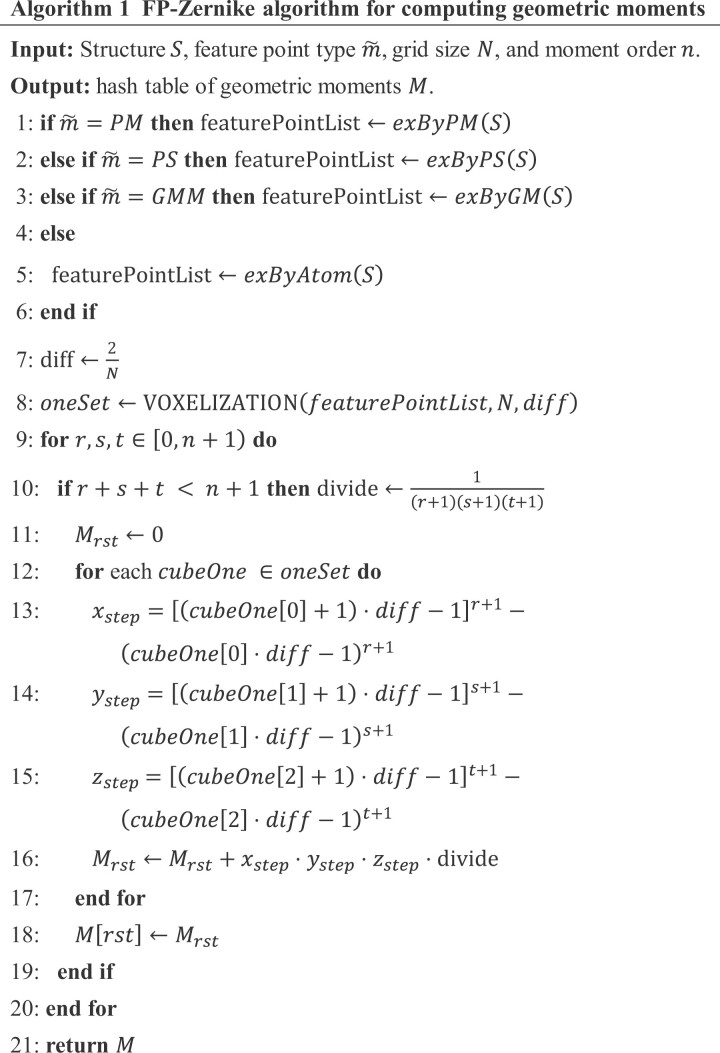

Algorithm 1 showed the process of computing geometric moments. Initially, it calculated the feature points of the structure (lines 1–6) based on the modes input by the user, utilizing four main functions: exByPM, exByPS, exByGM, and exByAtom, with detailed calculations provided in File S1 (see “Feature point extraction functions”). Subsequently, Algorithm 1 determined the size of each small cube in the grid according to the size of the grid (line 7) and calculated the small cubes in which the structure representation function took a value of 1 (line 9). Finally, the geometric moments were calculated according to Equation 5 (lines 10–21).

Algorithm 2 outlined the process of constructing a structural representation function f(X). Given *S*, the computational complexity of *f*(*x*,*y*,*z*) was *O*(|*S*|). It determined the indexes of the small cubes containing the feature points, where f(X) took a value of 1, and 0 in other cubes. Specifically, a collection named oneSet (Algorithm 2: line 2) was created to store the positions of all small cubes with a value of 1. Given a 3D point 3DPoint, the algorithm determined the index of the small cube into which it fell based on its three coordinate values (Algorithm 2: lines 4–6).

Upon calculating all geometric moments, the 3D Zernike descriptors could be obtained via Equations 2–4.

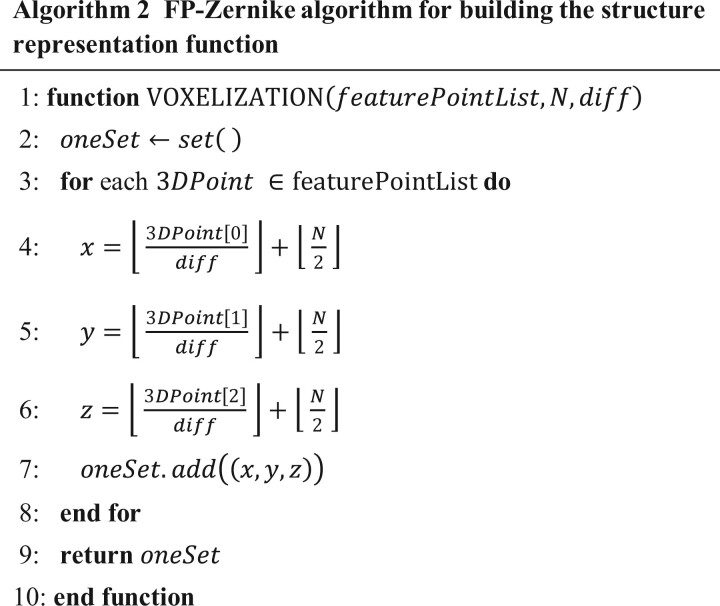

#### Build descriptor datasets based on FP-Zernike

A streamlined script was integrated into FP-Zernike, facilitating the rapid construction of descriptor datasets by users. With just a one-line command, users could calculate the descriptors for all structures within a folder containing files in .pdb format. The seamless integration underscores the ease with which FP-Zernike can be integrated into other structural analysis algorithms. The efficiency of building descriptor datasets is related to the complexity of the structure. The detailed information is provided in File S1 (see “Runtime analysis”).

#### Analysis of fusions of different representations

FP-Zernike can generate four distinct representations and descriptors for a given structure (Figure 1). By combining these representations using$\;C_{4}^{2}+C_{4}^{3}+C_{4}^{4}=11\;$fusion methods, new descriptors can be derived. For example, the “PM + PS” descriptor is obtained by combining the PM and PS representations. To generate the “PM + PS” descriptor, the PM and PS representations for a structure are initially computed. Subsequently, feature points are extracted from each representation and embedded into the unit ball, and then all feature points were used to generate the “PM + PS” descriptor. The process for generating new descriptors from other fused representations follows a similar approach.

We evaluated the performance of all the hybrid descriptors on the Protein160 dataset by clustering them. The results, summarized in **Table 1**, indicated that descriptors generated by a single representation outperformed those generated by a hybrid representation. Specifically, descriptors generated by the PM representation exhibited the best performance. These findings suggest that hybrid representations may not be as effective as single representations in representing structures and could potentially lead to “collision” issues, wherein these representations interfere with each other, diminishing the effectiveness of the mixed representation in representing the structure accurately.

**Table 1:** Clustering results obtained by different descriptors

Zernike mode	AMI	FMI	ACC	HOMO	COMP	V-measure
ATOM	0.82	0.70	0.79	0.91	0.93	0.92
GMM	0.61	0.45	0.60	0.81	0.84	0.82
PM	0.91	0.84	0.89	0.96	0.96	0.96
PS	0.85	0.75	0.83	0.93	0.94	0.93
ATOM + GMM	0.52	0.35	0.50	0.77	0.79	0.78
ATOM + PM	0.75	0.61	0.72	0.88	0.90	0.89
ATOM + PS	0.59	0.42	0.56	0.80	0.82	0.81
GMM + PM	0.75	0.62	0.72	0.88	0.90	0.89
GMM + PS	0.58	0.41	0.55	0.80	0.82	0.81
PM + PS	0.64	0.48	0.62	0.83	0.85	0.84
ATOM + GMM + PS	0.59	0.42	0.57	0.80	0.82	0.81
ATOM + GMM + PM	0.75	0.60	0.70	0.87	0.89	0.88
ATOM + PM + PS	0.64	0.48	0.62	0.83	0.85	0.84
GMM + PM + PS	0.64	0.47	0.61	0.82	0.85	0.83
ATOM + GMM + PM + PS	0.63	0.46	0.58	0.82	0.84	0.83

*Note*: Various representations were fused to generate descriptors on Protein160 and compute corresponding clustering metrics. V-measure represents the weighted average of HOMO and COMP. PS, protein surface; PM, protein mesh; GMM, Gaussian mixture model; ATOM, the atomic coordinates of the structure; AMI, adjusted mutual information; FMI, Fowlkes–Mallows index; ACC, accuracy; HOMO, homogeneity; COMP, completeness.

## Results

### Structure retrieval and classification by FP-Zernike effectively evaluate the structural similarity

FP-Zernike can compute four different Zernike descriptors based on four different representations for a given structure. To determine the most suitable descriptor for structure retrieval, we conducted experiments on benchmark datasets. The details on the evaluation metric can be found in the File S1 (see “Evaluation metric”).

We successfully obtained the descriptors of the Protein160 dataset through 3D-SURFER’s webserver. However, only 531 descriptors were attained for the Protein13 dataset using 3D-SURFER, resulting in a subset of Protein13 with the structures corresponding to these descriptors. Groups of smaller size (size < 5) in this subset were further excluded, yielding a dataset containing 529 structures from six groups, denoted by Protein13-Subset. We compared our method with 3D-SURFER and gmfit on Protein13-Subset. Since 3D-SURFER is not suitable for computing descriptors of RNA structures, our method was not compared with it on RNA16.

The top-*k* accuracy of our method and the two reference methods on Protein160, Protein13, Protein13-Subset, and RNA16 is shown in **Table 2**. Among the five modules of FP-Zernike, PM-Zernike was most stable and achieved superior results on three datasets, slightly surpassing gmfit and 3D-SURFER. GMM-Zernike and ReOmokage exhibited slightly lower top-10 accuracy on Protein160, but were outstanding on RNA16, likely due to their limited extraction of feature points and disregard for the internal structure information, leading to a slightly inferior performance on datasets containing complex structures. All methods demonstrated high top-*k* accuracy on Protein13, attributed to its small number of groups and the stringent criterion for determining membership, thereby simplifying the retrieval problem. Similarly, various methods also performed well on the Protein13-Subset. In particular, the top-*k* accuracy of FP-Zernike on RNA16 was relatively favorable compared with that of gmfit, indicating that FP-Zernike is more suitable for measuring the similarity between RNA structures.

**Table 2:** The top-*k* accuracy of our method and reference methods on the benchmark datasets

Dataset	Top-*k*	FP-Zernike component					Reference method	
		PM-Zernike	PS-Zernike	GMM-Zernike	ATOM-Zernike	ReOmokage	3D-SURFER	gmfit
Protein160	Top-10	**0.9114**	0.8540	0.6015	0.8378	0.5624	0.8762	0.9093
	Top-30	**0.9463**	0.8952	0.7148	0.8842	0.7364	0.9103	0.9237
	Top-50	**0.9540**	0.9160	0.7663	0.9026	0.8022	0.9238	0.9309
	Top-70	**0.9571**	0.9257	0.7916	0.9109	0.8438	0.9320	0.9341
	Top-100	**0.9596**	0.9365	0.8200	0.9299	0.8761	0.9465	0.9460
	Average accuracy	**0.9457**	0.9055	0.7388	0.8931	0.7642	0.9182	0.9288
Protein13	Top-10	**1.0000**	**1.0000**	0.9615	**1.0000**	**1.0000**	–	**1.0000**
	Top-30	0.9673	0.9628	0.9393	0.9825	0.9673	–	**0.9912**
	Top-50	0.9642	0.9592	0.9452	**0.9880**	0.9627	–	0.9672
	Top-70	0.9431	0.9284	0.9207	**0.9864**	0.9420	–	0.9536
	Top-100	0.9288	0.9304	0.9183	**0.9899**	0.9264	–	0.9451
	Average accuracy	0.9607	0.9562	0.9370	**0.9894**	0.9597	–	0.9714
RNA16	Top-10	0.8919	0.9200	**0.9263**	0.8441	0.9109	–	0.8326
	Top-30	0.8982	**0.9201**	0.9017	0.8818	0.9181	–	0.7571
	Top-50	0.9100	**0.9198**	0.8990	0.9097	0.9098	–	0.7495
	Top-70	0.9067	**0.9394**	0.9054	0.9066	0.9053	–	0.7440
	Top-100	0.9359	**0.9486**	0.9246	0.9075	0.9081	–	0.7442
	Average accuracy	0.9085	**0.9296**	0.9114	0.8899	0.9104	–	0.7655
Protein13-Subset	Top-10	**1.0000**	**1.0000**	**1.0000**	**1.0000**	**1.0000**	**1.0000**	**1.0000**
	Top-30	0.9444	0.9278	0.9444	**1.0000**	0.9278	0.9500	0.9056
	Top-50	**1.0000**	0.8967	0.9000	**1.0000**	0.8900	0.9033	0.8767
	Top-70	**1.0000**	0.8333	0.8810	**1.0000**	0.8785	**1.0000**	0.8643
	Top-100	**1.0000**	0.8683	0.8603	**1.0000**	0.8683	**1.0000**	0.8683
	Average accuracy	0.9889	0.9152	0.9187	1**.0000**	0.9129	0.9707	0.9030

*Note*: In each row of the table, the best value is bolded, and the next best value is underlined. –, not available.

**Figure 3:**
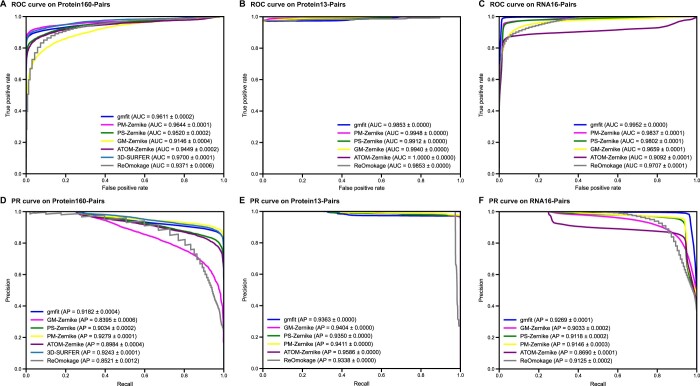
Comparison of structure retrieval performance among FP-Zernike, 3D-SURFER, and gmfit Comparison of ROC curves and AUC values for our method and the reference methods on the Protein160-Pairs (**A**), Protein13-Pairs (**B**), and RNA16-Pairs (**C**) datasets. Comparison of PR curves and AP values for our method and the reference methods on the Protein160-Pairs (**D**), Protein13-Pairs (**E**), and RNA16-Pairs (**F**) datasets. AUC and AP values are shown as mean ± standard deviation. ROC, receiver operating characteristic; AUC, area under the curve; PR, precision–recall; AP, average precision.

Furthermore, we evaluated the binary classification performance of FP-Zernike on structure pairs. **Figure 3** showed the receiver operating characteristic (ROC) and precision-recall (PR) curves for all methods. Overall, the binary classification accuracy of all methods was consistent with their top-*k* accuracy, with the exception of gmfit, which performed well in the binary classification of RNA16.

The aforementioned experiments indicate that PM-Zernike achieves the best performance, leading us to use it in building a retrieval system.

### FP-Zernike is generalized for a retrieve system on a large database

We acquired the complete Protein Data Bank (PDB) database (https://www.rcsb.org/), which contains 193,728 protein structures. Subsequently, all the structures were partitioned into single-stranded structures, yielding a large database of 590,685 structures. The descriptors (PM mode) for each structure were computed to construct the retrieval database. Based on this retrieval database, we built a retrieval system called FP-System. The workflow of the FP-System is as follows: the query structure is first verified for its presence in the database. If absent, its descriptor (referred to as the query descriptor) is calculated, followed by the computation of the Euclidean distance between all descriptors in the database and the query descriptor. The results are ranked based on the ascending order of the Euclidean distance (ascending order). Additionally, we offer a webserver to enhance the user-friendliness of FP-System, with detailed usage instructions available in File S1 (see “Instructions for use of the FP-Zernike”).

### FP-System holds superior suitability in structure retrieval

We utilized each structure in Random500 as a query structure and input them into 3D-SURFER and FP-System to obtain retrieval results. Here, we employed US-align to calculate the TM-score and the root-mean-square deviation (RMSD) between the structure in the retrieval results and the query structure, and used these two metrics to evaluate the performance of the retrieval system, with the main results presented in **Table 3**. The average TM-score of FP-System was 0.15–0.23 higher than that of 3D-SURFER, while the average RMSD of FP-System was ∼ 1 Å lower than that of the 3D-SURFER. These results indicate the superior suitability of FP-System for characterizing the structure compared with surface-based 3DZD.

**Table 3:** Retrieval evaluation of 3D-SURFER and FP-System on Random500

		Top-10	Top-30	Top-50	Top-100	Top-150
3D-SURFER	TM-score	0.606 ± 0.357	0.548 ± 0.350	0.522 ± 0.343	0.486 ± 0.331	0.464 ± 0.320
	RMSD (Å)	2.698 ± 2.253	3.133 ± 2.269	3.328 ± 2.252	3.595 ± 2.217	3.742 ± 2.172
FP-System	TM-score	0.835 ± 0.269	0.751 ± 0.314	0.708 ± 0.328	0.650 ± 0.337	0.614 ± 0.339
	RMSD (Å)	1.460 ± 1.879	2.086 ± 2.221	2.386 ± 2.326	2.812 ± 2.428	3.067 ± 2.465

*Note*: All values in the table were calculated by US-align, and represented by mean ± standard deviation. FP-System, FP-Zernike-based retrieval system; TM-score, template modeling score; RMSD, root-mean-square deviation.

### Real-time structural retrieval by FP-System

From the entire structure database, 1000 structure pairs were randomly selected, and aligned by TM-align, DeepAlign [33], US-align, gmfit, and FP-Zernike, respectively, to obtain the average alignment time. As shown in **Table 4**, FP-Zernike exhibited a very obvious efficiency advantage compared with other structural alignment tools. This might be owing to the reason that FP-Zernike measures the structural similarity by calculating the Euclidean distance between the descriptors of the structure, with the fixed descriptor length resulting in the time complexity of *O*(1).

**Table 4:** Average running time for the five tools to compute structural similarity

	FP-Zernike	gmfit	TM-align	DeepAlign	US-align
Running time (s)	2.75 × 10^−6^	0.710	0.038	0.221	0.078

### Efficient estimation of structural similarity based on Euclidean distance output from FP-Zernike

To facilitate a preliminary estimate of the similarity between structures based on Euclidean distance, we estimated the statistical significance of the Euclidean distance between FP-Zernike descriptors of structures by comparing approximately 1.99 million random structure pairs from our downloaded protein structure database (193,728 entries). GMM-Zernike was not included in this analysis due to its limited ability to characterize structures. A list of *P* values and corresponding FP-ATOM/FP-PM/FP-PS is provided in **Table 5**. For example, a FP-ATOM/FP-PM/FP-PS of 2.442935/3.932048/2.772549 indicated significant similarity at a *P* value of 0.01. These distance values for different Zernike descriptors can be utilized to rapidly estimate the similarity between structures.

**Table 5:** Statistical significance of the Euclidean distance between FP-Zernike descriptors of structures

Descriptor type	*P* value					
	5 × 10^−2^	1 × 10^−2^	1 × 10^−3^	1 × 10^−4^	1 × 10^−5^	1 × 10^−6^
FP-ATOM	2.987906	2.442935	1.988947	1.477029	0.522746	0.080739
FP-PM	4.653991	3.932048	3.169391	1.259989	0.252537	0.000244
FP-PS	3.299481	2.772549	2.306926	1.411444	0.514943	0.285697

## Discussion

In this study, we have developed an advanced toolkit named FP-Zernike, specifically designed for constructing structural descriptor databases. FP-Zernike enables the generation of four distinct representations and descriptors for structures. Extensive experiments have validated the effectiveness of these descriptors in capturing the characteristics of structures, particularly the PM-Zernike descriptor.

To showcase the capability of FP-Zernike, we constructed a PM-Zernike descriptor database tailored for protein structures at the chain level. Subsequently, we developed an ultra-fast retrieval system that leveraged this database. For a given query structure, our retrieval system can efficiently complete a structure retrieval within a remarkable 10-s timeframe. Experimental results further confirmed the high similarity between the retrieved structure and the query structure.

To facilitate easy access to the functionalities of FP-Zernike, we have integrated it into a user-friendly webserver. Users can conveniently utilize all the features of FP-Zernike by accessing it through the endpoint http://www.structbioinfo.cn/. Additionally, for users interested in analyzing customized datasets, the source code of FP-Zernike can be obtained through https://ngdc.cncb.ac.cn/biocode/tools/BT007365/releases/0.1 or https://github.com/junhaiqi/FP-Zernike.git.

By transforming structures into feature vectors, FP-Zernike can be applied to a wide range of structural analysis issues. Future endeavors will primarily focus on utilizing FP-Zernike to investigate protein–protein interactions. Specifically, FP-Zernike will be employed to convert proteins into feature vectors, which will then be integrated into neural networks to predict affinity between different structures.

## Conclusion

With the rapid growth of protein structure databases, an efficient and accurate structural similarity analysis scheme is urgently needed. Existing structure alignment algorithms cannot efficiently analyze the similarity between structures. In this work, we propose FP-Zernike, which allows users to compute different types of 3D Zernike descriptors and geometric features. Based on FP-Zernike, we built a retrieval system and demonstrated its superiority over the state-of-the-art retrieval system based on surface-based 3DZD. In terms of efficiency, FP-System can complete a retrieval in less than 10 s. In particular, users can easily deploy FP-Zernike in the local system for structure retrieval and descriptor database construction, as well as integrate it into other structure analysis algorithms.

## Code availability

All datasets and codes are available at https://ngdc.cncb.ac.cn/biocode/tools/BT007365/releases/0.1, as well as through a webserver at http://www.structbioinfo.cn/.

## CRediT author statement

**Junhai Qi:** Methodology, Software, Validation, Formal analysis, Writing – original draft, Visualization, Writing – review & editing. **Chenjie Feng:** Conceptualization, Investigation. **Yulin Shi:** Data curation, Formal analysis. **Jianyi Yang:** Conceptualization, Formal analysis. **Fa Zhang:** Conceptualization, Formal analysis. **Guojun Li:** Supervision, Investigation, Formal analysis, Resources. **Renmin Han:** Conceptualization, Investigation, Formal analysis, Supervision, Project administration, Writing – review & editing, Funding acquisition. All authors have read and approved the final manuscript.

## Supplementary material

Supplementary material is available at *Genomics, Proteomics & Bioinformatics* online (https://doi.org/10.1093/gpbjnl/qzae007).

## Competing interests

Junhai Qi was previously an intern at BioMap, and a portion of the work presented in this article was accomplished during his internship with the assistance of BioMap’s resources. All other authors have declared no competing interests.

## Supplementary Material

qzae007_Supplementary_Data

## References

[1] Jumper J , Evans R, Pritzel A, Green T, Figurnov M, Ronneberger O, et al Highly accurate protein structure prediction with AlphaFold. Nature 2021;596:583–9. 34265844 10.1038/s41586-021-03819-2 PMC8371605

[2] McLachlan AD. A mathematical procedure for superimposing atomic coordinates of proteins. Acta Crystallogr A 1972;28:656–7.

[3] Shindyalov IN , Bourne PE. Protein structure alignment by incremental combinatorial extension (CE) of the optimal path. Protein Eng 1998;11:739–47. 9796821 10.1093/protein/11.9.739

[4] Holm L , Sander C. Protein structure comparison by alignment of distance matrices. J Mol Biol 1993;233:123–38. 8377180 10.1006/jmbi.1993.1489

[5] Gong S , Zhang C, Zhang Y. RNA-align: quick and accurate alignment of RNA 3D structures based on size-independent TM-score_RNA_. Bioinformatics 2019;35:4459–61. 31161212 10.1093/bioinformatics/btz282 PMC6821192

[6] Zhang Y , Skolnick J. TM-align: a protein structure alignment algorithm based on the TM-score. Nucleic Acids Res 2005;33:2302–9. 15849316 10.1093/nar/gki524 PMC1084323

[7] Zhang C , Shine M, Pyle AM, Zhang Y. US-align: universal structure alignments of proteins, nucleic acids, and macromolecular complexes. Nat Methods 2022;19:1109–15. 36038728 10.1038/s41592-022-01585-1

[8] Kawabata T. Multiple subunit fitting into a low-resolution density map of a macromolecular complex using a Gaussian mixture model. Biophys J 2008;95:4643–58. 18708469 10.1529/biophysj.108.137125 PMC2576401

[9] Ljung F , André I. ZEAL: protein structure alignment based on shape similarity. Bioinformatics 2021;37:2874–81. 33772587 10.1093/bioinformatics/btab205 PMC10262298

[10] Bertolazzi P , Guerra C, Liuzzi G. A global optimization algorithm for protein surface alignment. BMC Bioinformatics 2010;11:488. 20920230 10.1186/1471-2105-11-488 PMC2957401

[11] Suzuki H , Kawabata T, Nakamura H. Omokage search: shape similarity search service for biomolecular structures in both the PDB and EMDB. Bioinformatics 2016;32:619–20. 26508754 10.1093/bioinformatics/btv614 PMC4743628

[12] Guzenko D , Burley SK, Duarte JM. Real time structural search of the Protein Data Bank. PLoS Comput Biol 2020;16:e1007970. 32639954 10.1371/journal.pcbi.1007970 PMC7371193

[13] Zhang Y , Sui X, Stagg S, Zhang J. FTIP: an accurate and efficient method for global protein surface comparison. Bioinformatics 2020;36:3056–63. 32022843 10.1093/bioinformatics/btaa076 PMC7214043

[14] Ellingson L , Zhang J. Protein surface matching by combining local and global geometric information. PLoS One 2012;7:e40540. 22815760 10.1371/journal.pone.0040540 PMC3398928

[15] Riahi S , Lee JH, Sorenson T, Wei S, Jager S, Olfati-Saber R, et al Surface ID: a geometry-aware system for protein molecular surface comparison. Bioinformatics 2023;39:btad196. 37067488 10.1093/bioinformatics/btad196 PMC10133531

[16] La D , Esquivel-Rodríguez J, Venkatraman V, Li B, Sael L, Ueng S, et al 3D-SURFER: software for high-throughput protein surface comparison and analysis. Bioinformatics 2009;25:2843–4. 19759195 10.1093/bioinformatics/btp542 PMC2912717

[17] Aderinwale T , Bharadwaj V, Christoffer C, Terashi G, Zhang Z, Jahandideh R, et al Real-time structure search and structure classification for AlphaFold protein models. Commun Biol 2022;5:316. 35383281 10.1038/s42003-022-03261-8 PMC8983703

[18] Sael L , Li B, La D, Fang Y, Ramani K, Rustamov R, et al Fast protein tertiary structure retrieval based on global surface shape similarity. Proteins 2008;72:1259–73. 18361455 10.1002/prot.22030

[19] Novotni M , Klein R. 3D Zernike descriptors for content based shape retrieval. Proc 8th ACM Symp Solid Model Appl 2003:216–25.

[20] Kawabata T. Gaussian-input Gaussian mixture model for representing density maps and atomic models. J Struct Biol 2018;203:1–16. 29522817 10.1016/j.jsb.2018.03.002

[21] Daberdaku S , Ferrari C. Antibody interface prediction with 3D Zernike descriptors and SVM. Bioinformatics 2019;35:1870–6. 30395191 10.1093/bioinformatics/bty918

[22] Daberdaku S , Ferrari C. Exploring the potential of 3D Zernike descriptors and SVM for protein–protein interface prediction. BMC Bioinformatics 2018;19:35. 29409446 10.1186/s12859-018-2043-3 PMC5802066

[23] Di Rienzo L , Milanetti E, Alba J, D’Abramo M. Quantitative characterization of binding pockets and binding complementarity by means of Zernike descriptors. J Chem Inf Model 2020;60:1390–8. 32050068 10.1021/acs.jcim.9b01066 PMC7997106

[24] Di Rienzo L , De Flaviis L, Ruocco G, Folli V, Milanetti E. Binding site identification of G protein-coupled receptors through a 3D Zernike polynomials-based method: application to *C. elegans* olfactory receptors. J Comput Aided Mol Des 2022;36:11–24. 34977999 10.1007/s10822-021-00434-1 PMC8831295

[25] Milanetti E , Miotto M, Di Rienzo L, Monti M, Gosti G, Ruocco G. 2D Zernike polynomial expansion: finding the protein-protein binding regions. Comput Struct Biotechnol J 2021;19:29–36. 33363707 10.1016/j.csbj.2020.11.051 PMC7750141

[26] Esquivel-Rodríguez J , Kihara D. Fitting multimeric protein complexes into electron microscopy maps using 3D Zernike descriptors. J Phys Chem B 2012;116:6854–61. 22417139 10.1021/jp212612t PMC3376205

[27] Venkatraman V , Yang YD, Sael L, Kihara D. Protein–protein docking using region-based 3D Zernike descriptors. BMC Bioinformatics 2009;10:407. 20003235 10.1186/1471-2105-10-407 PMC2800122

[28] Venkatraman V , Sael L, Kihara D. Potential for protein surface shape analysis using spherical harmonics and 3D Zernike descriptors. Cell Biochem Biophys 2009;54:23–32. 19521674 10.1007/s12013-009-9051-x

[29] Liao SX , Pawlak M. Image analysis with Zernike moment descriptors. CCECE’97. Canadian Conference on Electrical and Computer Engineering. Engineering Innovation: Voyage of Discovery. Conference Proceedings, St. John’s, NL, Canada 1997;2:700–3.

[30] Özbay E , Çinar A, Özbay FA. 3D human activity classification with 3D Zernike moment based convolutional, LSTM-deep neural networks. Trait Signal 2021;38:269–80.

[31] Johnson M , Zaretskaya I, Raytselis Y, Merezhuk Y, McGinnis S, Madden TL. NCBI BLAST: a better web interface. Nucleic Acids Res 2008;36:W5–9. 18440982 10.1093/nar/gkn201 PMC2447716

[32] Burley SK , Bhikadiya C, Bi C, Bittrich S, Chen L, Crichlow GV, et al RCSB Protein Data Bank: powerful new tools for exploring 3D structures of biological macromolecules for basic and applied research and education in fundamental biology, biomedicine, biotechnology, bioengineering and energy sciences. Nucleic Acids Res 2021;49:D437–51. 33211854 10.1093/nar/gkaa1038 PMC7779003

[33] Wang S , Ma J, Peng J, Xu J. Protein structure alignment beyond spatial proximity. Sci Rep 2013;3:1448. 23486213 10.1038/srep01448 PMC3596798

