# Supplementary material for: FP-Zernike: An Open-source Structural Database Construction Toolkit for Fast Structure Retrieval
Source: Genomics Proteomics Bioinformatics. 2024 Jan 19;22(1):qzae007. doi: 10.1093/gpbjnl/qzae007 (PMC11423855; doi:10.1093/gpbjnl/qzae007)
Supplement: qzae007_Supplementary_Data [file qzae007_supplementary_data.zip › TableS4-done.docx]

**Table S4 Performance evaluation of three metrics**

| **Dataset** | **Metric** | **Euclidean distance** | **Cosine similarity** | **Pearson correlation coefficient** | |
| --- | --- | --- | --- | --- | --- |
| Protein13-Test | AP | 0.93 | 0.93 | | 0.93 |
|  | AUC | 0.98 | 0.98 | | 0.98 |
| Protein160-Test | AP | 0.92 | 0.92 | | 0.92 |
|  | AUC | 0.97 | 0.97 | | 0.97 |
| RNA16-Test | AP | 0.90 | 0.90 | | 0.90 |
|  | AUC | 0.97 | 0.97 | | 0.97 |
