# Supplementary material for: FP-Zernike: An Open-source Structural Database Construction Toolkit for Fast Structure Retrieval
Source: Genomics Proteomics Bioinformatics. 2024 Jan 19;22(1):qzae007. doi: 10.1093/gpbjnl/qzae007 (PMC11423855; doi:10.1093/gpbjnl/qzae007)
Supplement: qzae007_Supplementary_Data [file qzae007_supplementary_data.zip › TableS5-done.docx]

**Table S5 Efficiency analysis of four modes of FP-Zernike**

|  | **Number of feature points** | **Average time (s)** | **Total time (s)** |
| --- | --- | --- | --- |
| ATOM | 575–7261 | 8.32 | 262.93 |
| PM | 4668–3,266,568 | 19.57 | 571.15 |
| PS | 14,136–6,009,687 | 23.78 | 681.53 |
| GMM | 606–29,252 | 45.09 | 1498.99 |

*Note*: Running the FP-Zernike on Random100 produced the statistics in the table. “Total time” represents the time it took FP-Zernike to generate all 3D Zernike descriptors (number of processes = 4).
