# Supplementary material for: FP-Zernike: An Open-source Structural Database Construction Toolkit for Fast Structure Retrieval
Source: Genomics Proteomics Bioinformatics. 2024 Jan 19;22(1):qzae007. doi: 10.1093/gpbjnl/qzae007 (PMC11423855; doi:10.1093/gpbjnl/qzae007)
Supplement: qzae007_Supplementary_Data [file qzae007_supplementary_data.zip › Figure S1.pdf]

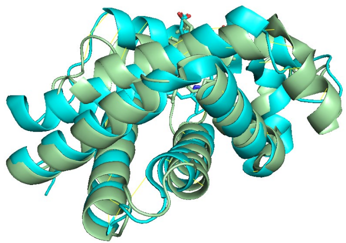

pymol-RMSD (1gdj, 1mbn) = 3.084

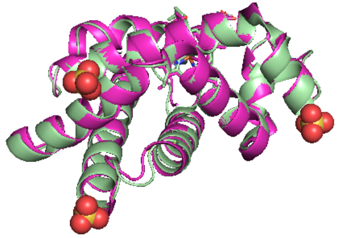

pymol-RMSD (1jw8, 1mbn) = 0.504

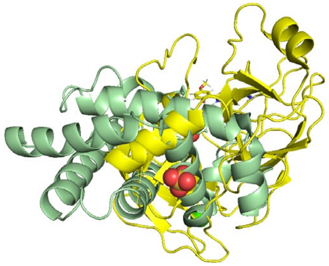

pymol-RMSD (1gi5, 1mbn) = 10.719

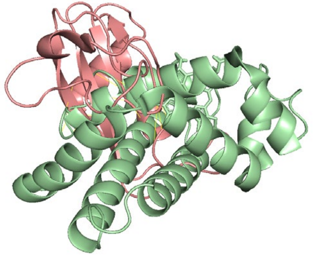

pymol-RMSD (1ss6, 1mbn) = 4.230

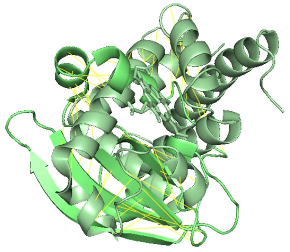

pymol-RMSD (1ywx, 1mbn) = 11.045

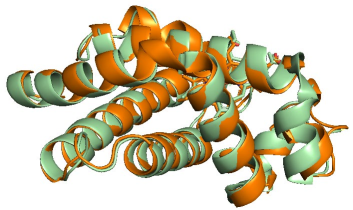

pymol-RMSD (1lhs, 1mbn) = 0.639

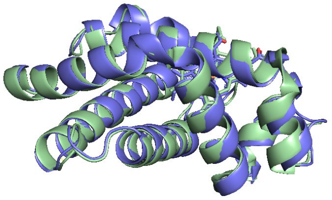

pymol-RMSD (1lht, 1mbn) = 0.642

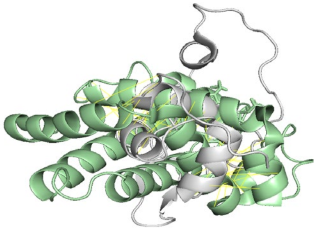

pymol-RMSD (1v4r, 1mbn) = 4.230

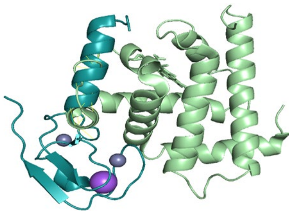

pymol-RMSD (3eb5, 1mbn) = 5.099

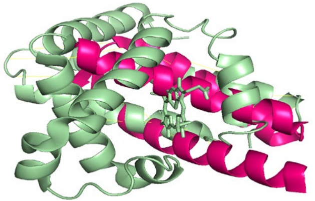

pymol-RMSD (2oo2, 1mbn) = 7.640
