# Supplementary material for: FP-Zernike: An Open-source Structural Database Construction Toolkit for Fast Structure Retrieval
Source: Genomics Proteomics Bioinformatics. 2024 Jan 19;22(1):qzae007. doi: 10.1093/gpbjnl/qzae007 (PMC11423855; doi:10.1093/gpbjnl/qzae007)
Supplement: qzae007_Supplementary_Data [file qzae007_supplementary_data.zip › Figure S4.pdf]

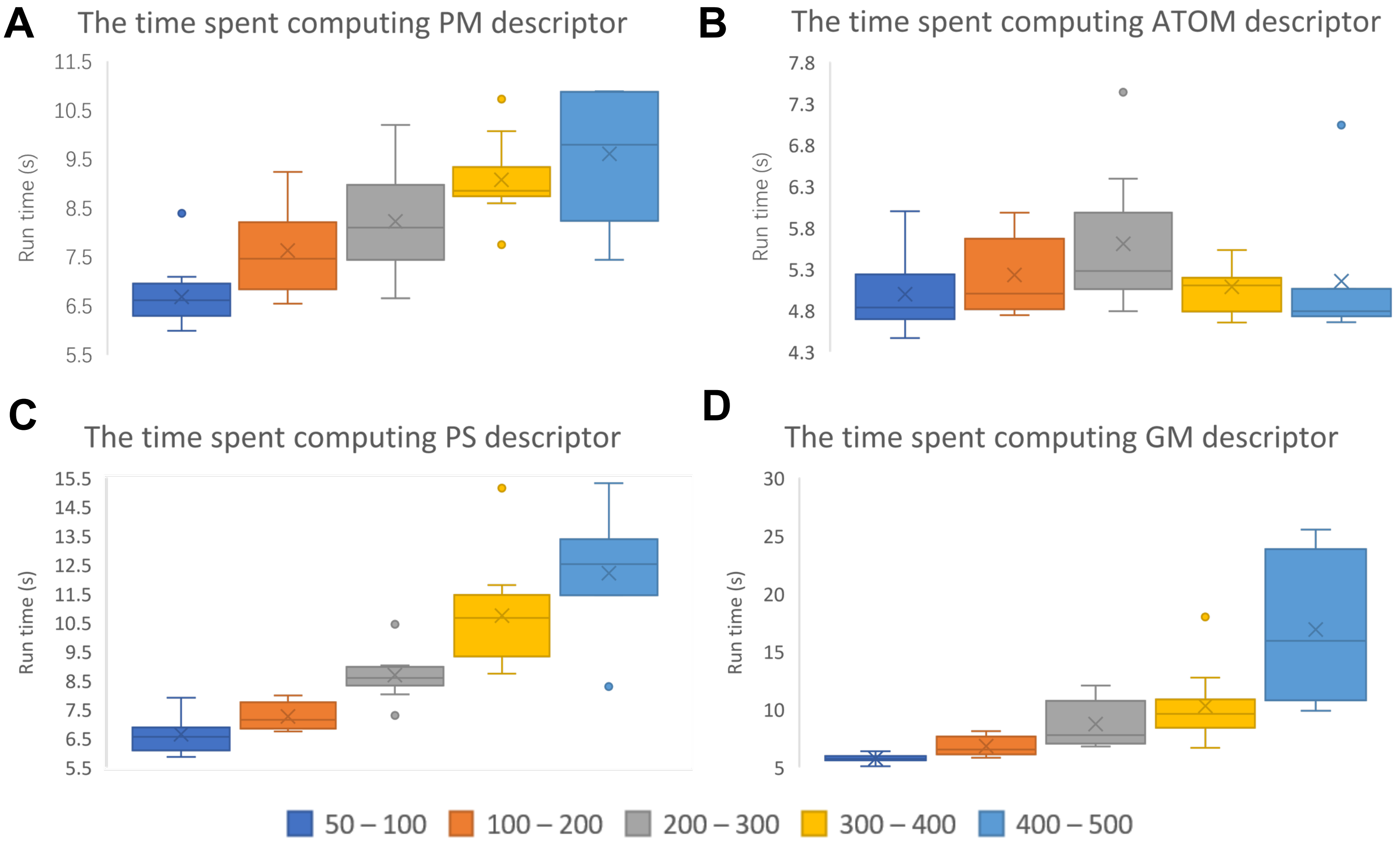

A
The time spent computing PM descriptor
Run time (s)
11.5
10.5
9.5
8.5
7.5
6.5
5.5
B
The time spent computing ATOM descriptor
Run time (s)
7.8
7.3
6.8
6.3
5.8
5.3
4.8
4.3
C
The time spent computing PS descriptor
Run time (s)
15.5
14.5
13.5
12.5
11.5
10.5
9.5
8.5
7.5
6.5
5.5
D
The time spent computing GM descriptor
Run time (s)
30
25
20
15
10
5
50 – 100
100 – 200
200 – 300
300 – 400
400 – 500
