# Supplementary material for: FP-Zernike: An Open-source Structural Database Construction Toolkit for Fast Structure Retrieval
Source: Genomics Proteomics Bioinformatics. 2024 Jan 19;22(1):qzae007. doi: 10.1093/gpbjnl/qzae007 (PMC11423855; doi:10.1093/gpbjnl/qzae007)
Supplement: qzae007_Supplementary_Data [file qzae007_supplementary_data.zip › Figure S5.pdf]

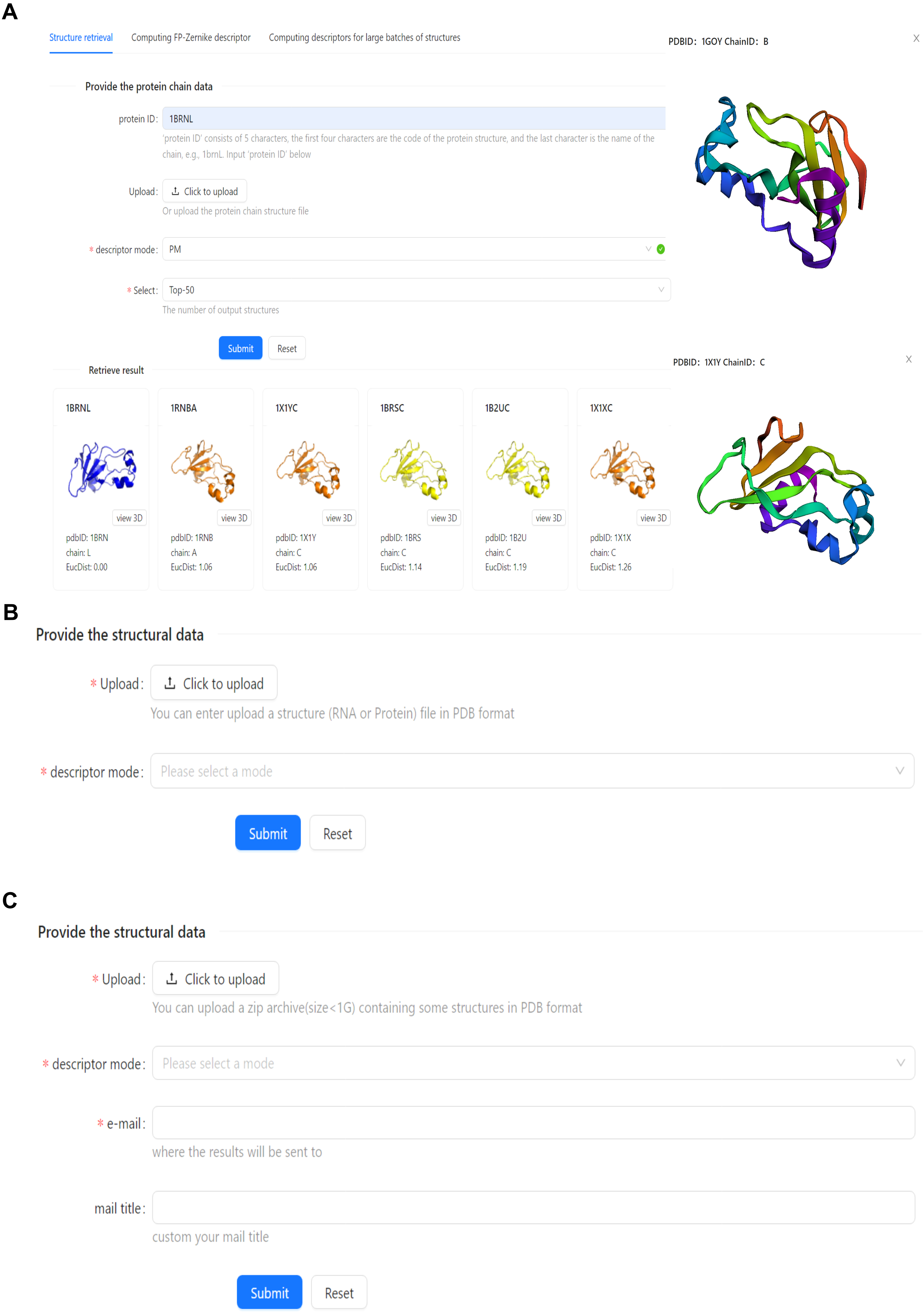
A
Structure retrieval
Computing FP-Zernike descriptor
Computing descriptors for large batches of structures
PDBID: 1GOY ChainID: B
Provide the protein chain data
protein ID: 1BRNL
'protein ID' consists of 5 characters, the first four characters are the code of the protein structure, and the last character is the name of the chain, e.g., 1brnL. Input 'protein ID' below
Upload: Click to upload
Or upload the protein chain structure file
* descriptor mode: PM
* Select: Top-50
The number of output structures
Submit
Reset
Retrieve result
PDBID: 1X1Y ChainID: C
1BRNL
1RNBA
1X1YC
1BRSC
1B2UC
1X1XC
view 3D
pdbID: 1BRN
chain: L
EucDist: 0.00
pdbID: 1RNB
chain: A
EucDist: 1.06
pdbID: 1X1Y
chain: C
EucDist: 1.06
pdbID: 1BRS
chain: C
EucDist: 1.14
pdbID: 1B2U
chain: C
EucDist: 1.19
pdbID: 1X1X
chain: C
EucDist: 1.26
B
Provide the structural data
* Upload: Click to upload
You can enter upload a structure (RNA or Protein) file in PDB format
* descriptor mode: Please select a mode
Submit
Reset
C
Provide the structural data
* Upload: Click to upload
You can upload a zip archive(size<1G) containing some structures in PDB format
* descriptor mode: Please select a mode
* e-mail:
where the results will be sent to
mail title:
custom your mail title
Submit
Reset
