# Supplementary material for: FP-Zernike: An Open-source Structural Database Construction Toolkit for Fast Structure Retrieval
Source: Genomics Proteomics Bioinformatics. 2024 Jan 19;22(1):qzae007. doi: 10.1093/gpbjnl/qzae007 (PMC11423855; doi:10.1093/gpbjnl/qzae007)
Supplement: qzae007_Supplementary_Data [file qzae007_supplementary_data.zip › FileS1-done.docx]

**File S1 Supplementary materials**

**Analysis of moments and 3D zernike moments**

*The definition of the moments*

Moment is a scalar used to characterize and capture important features of a function. The standard expression of moment can be referred to Equation S1.

$$\begin{aligned} \mu_{i}:\text{ }<f\left( X \right),\varphi_{i}\left( X \right)\geq\int_{\Omega} f\left( X \right)\cdot\hat{\varphi_{i}\left( X \right)}dX\#\left( S1 \right) \end{aligned}$$

Here, $f(X)$ is a square integrable function defined on $\Omega$, $\psi=\{\varphi_{i}\}$ is the set of functions, and $\hat{\varphi_{i}\left( X \right)}$ is the conjugate function of $\varphi_{i}(X)$.

When $\psi$ is given, the moments with respect to $f(X)$ are completely determined, so the $\psi$ completely determines the properties of moments. Going through all the functions in $\psi$ and substituting them into Equation S1, we get a vector $\mu=\left( \mu_{1},\mu_{2},\ldots,\mu_{\left| \psi\right|} \right)$, and $\mu$ is called the descriptor of the function with respect to moments.

*Moments with good properties*

When $f(X)$ represents a 3D structure, we expect that descriptor generated from moments has some good properties. For example, scale invariance, translation invariance, and rotation invariance. These invariance properties should be satisfied because the basic form of the 3D structure remains the same no matter how it is rotated, scaled, or translated.

On the other hand, we need to put some restrictions on $\psi$. For $\forall\varphi_{i}\left( X \right),\varphi_{i}\left( X \right)\in\psi$, Equation S2 should be satisfied, which implies that the information of descriptor generated by $\psi$ is non-redundant.

$$\begin{aligned} <\varphi_{i}\left( X \right),\varphi_{j}\left( X \right)\geq\left\{ \begin{matrix} 1, & \text{if }i=j \\ 0, & \text{otherwise } \end{matrix} \right.\#\left( S2 \right) \end{aligned}$$

In addition, we hope that descriptor strongly correlated with $f(X)$, that is, $f(X)$ can be reconstructed by the descriptor $\mu$. Therefore, Equation S3 should be satisfied.

$$\begin{aligned} \lim_{n\to\infty}||f(X)-\sum_{i=0}^{n} <f(X),\varphi_{i}(X)>\varphi_{i}(X)||^{2}=0\#\left( S3 \right) \end{aligned}$$

*3D Zernike descriptor*

Equation S4 defines the 3D Zernike moments. The descriptor induced by 3D Zernike moments is called 3D Zernike descriptor, which satisfies the good properties mentioned above.

$$\begin{aligned} \Omega_{nl}^{m}:=\frac{3\pi}{4}\int_{\Omega} f\left( X \right)\hat{Z_{nl}^{m}\left( X \right)}dX\#\left( S4 \right) \end{aligned}$$

Here, $Z_{nl}^{m}$ is defined in Equation S5, where $n,l,m$ are integers, $l\in[0,n]$ and $n-l$ is even, $m\in[-l,l]$ and $X\in\mathbb{R}^{3}$.

$$\begin{aligned} Z_{nl}^{m}\left( X \right):=\sum_{v=0}^{k} q_{kl}^{v}\left| X \right|^{2v}e_{l}^{m}\left( X \right)\#\left( S5 \right) \end{aligned}$$

In addition, the definition of $q_{kl}^{v}$ can be found in Equation S6, and the definition of $e_{l}^{m}(X)$can be found in Equation S7.

$$\begin{aligned} q_{kl}^{v}=\frac{(-1)^{k}}{4^{k}}\sqrt{\frac{2l+4k+3}{3}}\binom{2k}{k}(-1)^{v}\cdot\frac{({k \atop v})({2(k+l+v)+1 \atop2k})}{({k+l+v \atop k})}\#\left( S6 \right) \end{aligned}$$

$$\begin{aligned} e_{l}^{m}(X)=c_{l}^{m}|X|^{l}(\frac{ix-y}{2})^{m}z^{l-m}\cdot\sum_{\mu=0}^{\frac{\lfloor l-m\rfloor}{2}} \binom{l}{\mu}\binom{l-\mu}{m+\mu}(-\frac{x^{2}+y^{2}}{4z^{2}})^{\mu}\#\left( S7 \right) \end{aligned}$$

Here, $X=(x,y,z)$, $i$ is imaginary number and $c_{l}^{m}$ is defined in Equation S8.

$$\begin{aligned} c_{l}^{m}=c_{l}^{-m}=\frac{\sqrt{\left( 2l+1 \right)\left( l+m \right)!\left( l-m \right)!}}{l!}\#\left( S8 \right) \end{aligned}$$

We define $\Omega_{nl}=\left( \Omega_{nl}^{-l},\Omega_{nl}^{-l+1},\ldots,\Omega_{nl}^{l} \right)$, $F_{n,l}=\left| \Omega_{nl} \right|$, and then the 3D Zernike descriptor ($order=n$) of $f\left( X \right)$ is defined as the following vector $D_{f}$(Equation S9).

$$\begin{aligned} D_{f}=\left( F_{n,l_{0}},F_{n,l_{1}},\ldots,F_{n,l_{t}} \right)\#\left( S9 \right) \end{aligned}$$

Here, $n-l_{i}$ is even and $l_{i}\in\left[ 0,n \right]$($0 \leq i \leq t < n$).

**Feature point extraction functions and benchmark construction**

*Feature point extraction functions*

When generating the structure representation file, we employed four feature point extraction functions: $\mathrm{exByPM}$, $\mathrm{exByPS}$, $\mathrm{exByGM}$, and $\mathrm{exByAtom}$, each corresponding to a specific mode of extraction. The format of the structure representation file follows the sequence: $\mathrm{wrl}$, $\mathrm{exByPM}$, $\mathrm{exByPS}$, and $\mathrm{exByGM}$ for extracting feature points from the representation file, while $\mathrm{exByAtom}$ for retrieving atomic coordinates from the $\mathrm{pdb}$ file.

*Benchmark datasets construction*

Protein160. We carefully constructed this dataset based on basic local alignment search tool [1], farthest point sampling-enhanced triangulation-based iterative-closest-point (FTIP) , and TM-align, and controlled the size of the structures in the dataset and the size of the groups. The dataset consists of 1552 protein chains with the sequence length between 93 and 200 amino acid residues. All protein chains are divided into 160 groups with 7–9 elements in each groups. Here, intra-group sequence identity > 0.9 and inter-group sequence identity < 0.1. Additionally the conditions are met: TM-score > 0.8 between structures in the same group, TM-score < 0.3 between structures in different groups.

Protein13. We used 13 protein structures with differences as template structures, obtained the retrieval results based on web server [2], and then further screened based on TM-align, and finally determined this dataset. The dataset consists of 1431 protein structures with the sequence length between 114 and 584 amino acid residues. We do not control the size of the groups, and each group has 20–254 structures, making a total of 13 groups and the other conditions are met: TM-score > 0.85 between structures in the same group, TM-score < 0.3 between structures in different groups.

RNA16. In our downloaded RNA structure database (11,663 entries), we randomly selected a structure as the query structure (representative element, denoted by $q$), and used RNA-align to screen out the structures (denoted by $E$) that meet certain conditions (for $\forall e \in E$, TM-score$\left( q,e \right)>0.8$ and $r\mathrm{oot}m\mathrm{ean}s\mathrm{quare}d\mathrm{eviation}\left( q,e \right)<6 (RMSD<6)$). These structures are considered similar to query structure. We iteratively selected query structures and generated groups corresponding to the query structures. Let $Q$ be the set of all query structures, for any structure $e$ in the group $g$, if it satisfies certain conditions ($\exists q_{1}\in Q,q_{2}\in Q,s.t.\mathrm{TM}-\mathrm{score}\left( q_{1} \left( q_{2} \right),e \right)>0.8$ or $RMSD\left( q_{1} \left( q_{2} \right),e \right)<6$), it will be eliminated. Finally, the dataset consists of 955 RNA structures with sequence lengths ranging from 33 bp to 4010 bp, divided into 16 groups of 7–342 structures.

Structure-Pairs. We obtained all structural pairs for each dataset and classified them into positive and negative samples. Here, if two structures in a structure pair belong to the same group, the structure pair is a positive sample, otherwise it is a negative sample. Finally, Protein160 generated 6722 positive samples and 1,198,406 negative samples, Protein13 generated 130,671 positive samples and 893,925 negative samples, and RNA16 generated 83,839 positive samples and 372,651 negative samples. We used random sampling to deal with the problem of excessive imbalance in the number of positive and negative samples. Specifically, we randomly sampled three times the number of positive samples among all negative samples, so each dataset produced 3 negative sample datasets. All positive and negative datasets are used to evaluate the binary classification performance of FP-Zernike.

Random100. In our downloaded protein structure database (193,728 entries), we randomly selected 100 structures (the number of atoms is greater than 500) to build this dataset in order to test the efficiency of FP-Zernike.

*Reference methods*

3D-SURFER 3D-SURFER is a web server that primarily focuses on structure retrieval. It employs a precomputation approach, generating a vast collection of 3D Zernike descriptors for protein structures, which are then stored in a database. Users can readily access these precomputed 3D Zernike descriptors. However, when it comes to user-defined datasets, relying on 3D-SURFER for timely computation of 3D Zernike descriptors may not be feasible. In your specific case, despite submitting compressed files of RNA structures in the required format, the computation of 3D Zernike descriptors will be unsuccessful. Therefore, it may not be possible to rely on 3D-SURFER to obtain the 3D Zernike descriptors for user-defined datasets within a reasonable timeframe.

gmfit. gmfit is a structural alignment tool, which is different from other alignment tools in that it completes the alignment process based on the Gaussian mixture model of the structure. We can calculate the Gaussian mixture model of the structure in advance to build the retrieval system, so here we chose it for comparison with FP-Zernike. Taking 3o2z.pdb and 3o58.pdb as an example, the following is the command line when we use gmfit to complete the comparison process:

Obtain the Gaussian mixture model of the structure:

**gmconvert -ipdb 3o2z.pdb -ng 50 -maxsize 64 -ogmm 3o2z_g50.gmm**

**gmconvert -ipdb 3o58.pdb -ng 50 -maxsize 64 -ogmm 3o58_g50.gmm**

Aligning based on Gaussian mixture model:

**gmfit -cg 3o2z\_g50.gmm -sg1 3o58\_g50.gmm -I P -ochimera T**

**Experimental details for the comparison of ReOmokage and Omokage**

*ReOmokage*

ReOmokage is similar to Omokage, and compared with Omokage, ReOmokage has two main differences: (1) Based on quanpdb [3], ReOmokage may uses different parameters to extract the feature points of the structure. (2) Omokage only provides web services, while ReOmokage is a localized algorithm that allows users to calculate the similarity between their customized structures. The process by which ReOmokage calculates the similarity between two structures is as follows. First, ReOmokage extracts feature points about two structures based on quanpdb. Here, we simply tunes quanpdb (with some parameters fixed for automation). The parameter usage of quanpdb may be different from Omokage (Omokage does not disclose the usage details of quanpdb). Then, based on the feature points, the distance distribution is calculated and the incremental distance rank (IDR) profiles are generated. Finally, a well-designed metric is used to calculate the similarity between IDR profiles to measure the similarity between structures.

*1084 structure pairs and corresponding Omokgae scores*

In order to evaluate the structural similarity between protein structures, we conducted a comparison between ReOmokage and Omokage. However, it is important to note that Omokage scores are not readily available for arbitrary structures. To overcome this limitation, we selected a query structure (1mbn.pdb) and obtained the query results from the Omokage server (https://pdbj.org/omokage?lang=zh-CN).

The query results provided us with Omokage scores between the query structure and a set of 2000 structures. From this dataset, we obtained 1084 structure files in pdb format, along with their corresponding Omokage scores. To further analyze the structural similarity, we employed ReOmokage to calculate ReOmokage scores between the query structure and the aforementioned 1084 structures.

*ReOmokage vs. Omokage*

We conducted an analysis to compare the differences between ReOmokage scores and Omokage scores for a set of 1084 structural pairs. Among these pairs, we found that 31 of them exhibited significant differences, with a discrepancy greater than 0.1 between the Omokage score and the ReOmokage score.

To evaluate the performance of both scores on these structural pairs, we utilized TM-score and pymol-RMSD as gold standards. Table S3 presents the values of the Omokage score and the ReOmokage score for these structural pairs. In the table, a $\mathrm{Shot}$ value of 1 indicates that the ReOmokage score is more reasonable, closer to the TM-score and pymol-RMSD. Additionally, Figure S1 displays the visual representations of 10 structural pairs.

Based on the information provided in Table S3, we observed that the ReOmokage score provides a more reasonable assessment for 22 out of the 31 structural pairs, resulting in a success rate of approximately 0.7096 (22/31).

*Difference analysis between ReOmokage and Omokage*

We conducted a detailed analysis to investigate the reasons behind the differences between ReOmokage and Omokage scores for the ten most divergent structural pairs. By examining Figure S2, we observed that the feature points of the 1mbn.pdb structure, as determined by ReOmokage, are significantly distinct from those of the other structures. This discrepancy helps explain why ReOmokage assigns lower scores to these structural pairs.

**FP-system**

*Evaluation metric*

The calculation formula of top-*k* accuracy is as follows:

$$\begin{aligned} top-k\text{ }accuracy=\frac{K\cap C}{min\left( k,\left| C \right| \right)}\#\left( 10 \right) \end{aligned}$$

Here, $k$ represents the set consisting of the first $k$ items in the query result, and $C$ represents the set consisting of structures similar to the query structure. Two metrics are used to evaluate the binary classification results, namely area under curve (AUC) and average precision (AP). Here, AUC is the area under the receiver operating characteristic (ROC), and AP is the area under the precision–recall (PR) curve. The larger the values of AUC and AP, the better the classification effect of the model.

*Analysis of similarity metrics*

After converting protein structures into FP-Zernike descriptors, the next step is to measure the similarity between descriptors, which reflects the similarity between structures. Three metrics were evaluated for this purpose: Euclidean distance, cosine similarity, and Pearson correlation coefficient. To assess the performance of these metrics, three test sets were created by randomly selecting 1000 positive and 2000 negative samples from Protein160-Pairs, Protein13-Pairs, and RNA16-Pairs, respectively. These test sets were named Protein160-Test, Protein13-Test, and RNA16-Test.

Using the PM-Zernike descriptor, the binary classification performance of the three metrics was evaluated, and the results are shown in Table S4. The performance of the three metrics was found to be identical, indicating that they are equivalent. For simplicity, the Euclidean distance was chosen as the metric for measuring the similarity between descriptors.

*Runtime analysis*

We mainly focus on the computational efficiency of the 3D Zernike descriptors of the FP-Zernike, because the efficiency of constructing geometric features with Reomokage mainly depends on situs. Here, We tested the efficiency of FP-Zernike based on Random100. Table S5 shows the main information generated by building the database of 3D Zernike descriptors. We can see that the Atomic (ATOM) mode of FP-Zernike has the highest efficiency in computing 3D Zernike descriptors, and 3D Zernike descriptors of 100 structures can be calculated in 263s. The efficiency of pymol surface (PS) mode and pymol mesh (PM) mode is comparable, and the efficiency of Gaussian mixture model (GMM) mode is the lowest. We can infer that with sufficient computational resources (16 processes), we can construct the 3D Zernike descriptor dataset for the entire protein structure database (<https://www.rcsb.org/>) in less than 1 day. In addition, we can from Table S5 see that the PM mode and PS mode of FP-Zernike have the largest number of feature points, which implies that the 3D Zernike descriptors generated in these two modes may contain more sufficient information.

We also analyzed the influencing factors of FP-Zernike efficiency. Figure S3 presents the main results, and we can see that two key factors (number of feature points and the efficiency of building a structural representation) that are linearly related to the efficiency of FP-Zernike. In addition, we analyzed the running time ratio of each module of the four modes of FP-Zernike, we can see that the time spent on each module for computing 3D Zernike descriptors is similar in PS, PM, and ATOM modes. In GMM mode, the main running time lies in the computation of the structural representation. On the other hand, we explored the relationship between the computational efficiency of FP-Zernike and the size of the structure, and Figure S4 shows the main results. From the figure, we can see that the calculation efficiency of FP-Zernike and the size of the structure are roughly a linear negative correlation. This is consistent with Figure S3A, since the larger the size of the structure, the more feature points generated by it.

Combining the efficiency analysis of each mode of FP-Zernike and the retrieval accuracy, PM, PS, and ATOM are the recommended modes for building a database of 3D Zernike descriptors..

**Instructions for use of the FP-Zernike**

*Packages and deploy*

FP-Zernike is a python package that can be quickly deployed on linux systems. Users need to use the command line to install FP-Zernike in the terminal. In addition, the installation needs to rely on Conda. If the user does not have Conda, use the following command line (font has been bolded) to install.

1. Download Anaconda3

**wget** [**https://mirrors.tuna.tsinghua.edu.cn/anaconda/archive/Anaconda3-2021.11-Linux-x86_64.sh**](https://mirrors.tuna.tsinghua.edu.cn/anaconda/archive/Anaconda3-2021.11-Linux-x86_64.sh)

1. Install Conda

**bash Anaconda3-2021.11-Linux-x86_64.sh**

When Conda is successfully installed, the user only needs to enter the following commands to deploy the FP-Zernike.

1. Download FP-Zernike from *github*

**git clone** [**https://github.com/junhaiqi/FP-Zernike.git**](https://github.com/junhaiqi/FP-Zernike.git)

1. Enter the folder

**cd FP-Zernike-main**

1. Create a folder

**mkdir tempoutput**

1. Open permissions for some tools

**chmod 777 tool/gmconvert**

**chmod 777 tool/extractFeaturePoints30**

**chmod 777 tool/extractFeaturePoints50**

1. Deploy

**conda env create -f PGAR_Zernike.yaml**

Users can use the following commands to test whether FP-Zernike can be used normally.

1. Activation environment and install a package

**source activate PGAR_Zernike**

**pip install scikit-learn**

1. Test

**python mainZernikeCalculation.py -i example/4mu3A.pdb -file_source pymol -mode mesh -o test_4mu3A_PM.pkl**

If the above command can be run successfully, then FP-Zernike has been successfully deployed.

*Usage*

Given the query structure (path: example/4mu3A.pdb), the user can execute the following command to get its ATOM-Zernike:

**python mainZernikeCalculation.py -i example/4mu3A.pdb -file_source atom -mode atom -o test_4mu3A_ATOM.pkl**

Here, “test_4mu3A_ATOM.pkl” include the information of Zernike descriptor of “example/4mu3A.pdb”.

The user can execute the following command to get its PM-Zernike:

**python mainZernikeCalculation.py -i example/4mu3A.pdb -file_source pymol -mode mesh -o test_4mu3A_PM.pkl**

The user can execute the following command to get its PS-Zernike:

**python mainZernikeCalculation.py -i example/4mu3A.pdb -file_source pymol -mode surface -o test_4mu3A_PS.pkl**

The user can execute the following command to get its GMM-Zernike:

**python mainZernikeCalculation.py -i example/4mu3A.pdb -file_source gmconvert -mode mesh -o test_4mu3A_GM.pkl**

In addition, users can enter the following command to obtain detailed instructions.

**python mainZernikeCalculation.py -h**

The above commands are in “test_mainZernikeCalculation.sh”, and user can run “bash test_mainZernikeCalculation.sh” to test this function.

*Build a database of descriptors*

Given the customized dataset (only include *.pdb file, path: example), the user can execute the following command to get its ATOM-Zernike database:

**python mainMakeZernikeDataBase.py -i example -t 4 -mode ATOM**

Here, “4” is the thread number. A new folder (path: exampleAtomZernikeDescriptor) containing all descriptors is automatically generated. Similarly, descriptors for other modes can also be built, and these command lines are in “test_mainMakeZernikeDataBase.sh” user can run “**bash test_mainMakeZernikeDataBase.sh**” to test this function. In addition, users can enter the following command to obtain detailed instructions.

**python mainRetrieve.py -h**

*Ultra-fast structure retrieval*

Given the query structure, the user can perform ultra-fast structure retrieval. For example, given a protein chain (Path: example/4mu1A.pdb), users can preform sturcture retrieval by command as follows.

**python mainRetrieve.py -q example/4mu1A.pdb -dm PM -otn 10 -op testPMRtriRes.txt**

Here, “PM” is the mode of retrieve, “10” is the number of output structures, “testPMRtriRes.txt” stores all the output.

In addition, users can enter the following command to obtain detailed instructions.

**python mainRetrieve.py -h**

There are more commands in “test_mainRetrieve.sh”, and users can test this function by executing “bash test_mainRetrieve.sh”. We constructed a demo dataset containing 590,685 structures. At this scale, our retrieval system only takes 4–9 s to complete a retrieval.. To perform the retrieval on the demo dataset, users need to through the following link to download demo database (Name: PMAllSingleChainZernike.pkl), and need to put “PMAllSingleChainZernike.pkl” copy to the folder “database”.

**link:** [**https://pan.baidu.com/s/1NnQsFiVyatQ2p-k7oj-giA**](https://pan.baidu.com/s/1NnQsFiVyatQ2p-k7oj-giA)

**Extraction code: 208z**

*Measure the similarity between the two structures*

When the descriptors of the structure are calculated by FP-Zernike, the user can initially measure the similarity based on the Euclidian distance between the descriptors. Specifically, given the descriptors of two structures, A (Path: example/4mr5A.pkl) and B (Path: example/4mr6A.pkl), the user can perform the following command to obtain the Euclidean distance between them.

**python mainZernikeDist.py example/4mr5A.pkl example/4mr6A.pkl**

For the relationship between Euclidean distance value and similarity, users can refer to Table 5 in the main text.

*Measure the similarity based on ReOmokage*

Here, we improved the Omokage and proposed ReOmokage to measure the similarity between structures. Given structures A (Path: example/4mu3A.pdb) and B (Path: example/4mu1A.pdb), the user can enter the following command to calculate the similarity between the structures:

**python mainReOmokage.py -pdb1 example/4mu3A.pdb -pdb2 example/4mu1A.pdb**

*A webserver*

We offered a webserver to enhance the usability of FP-Zernike for users. It provides a user-friendly interface (see Figure S5) that enables users to retrieve protein structures and generate different types of descriptors with ease.

**References**

1. Johnson M, Zaretskaya I, Raytselis Y, Merezhuk Y, McGinnis S, Madden TL. NCBI BLAST: a better web interface. Nucleic Acids Res 2008;36:W5−9.
2. Burley SK, Bhikadiya C, Bi C, Bittrich S, Chen L, Crichlow GV, et al. RCSB Protein Data Bank: powerful new tools for exploring 3D structures of biological macromolecules for basic and applied research and education in fundamental biology, biomedicine, biotechnology, bioengineering and energy sciences. Nucleic Acids Res 2021;49:D437−51.
3. Wriggers W, Milligan RA, McCammon JA. Situs: a package for docking crystal structures into low-resolution maps from electron microscopy. J Struct Biol 1999;125:185−95.
