# Supplementary material for: FP-Zernike: An Open-source Structural Database Construction Toolkit for Fast Structure Retrieval
Source: Genomics Proteomics Bioinformatics. 2024 Jan 19;22(1):qzae007. doi: 10.1093/gpbjnl/qzae007 (PMC11423855; doi:10.1093/gpbjnl/qzae007)
Supplement: qzae007_Supplementary_Data [file qzae007_supplementary_data.zip › Supplementary captions.docx]

**Supplementary material**

**File S1** **Supplementary materials**

**Figure S1 Ten conformations resulting from the superposition of 10 structures with 1mbn.pdb and the corresponding pymol-****RMSD (**$\mathbf{Å}$**)**

The “1lhs-1mbn” represents the conformation resulting from the superposition of the structure 1lhs.pdb and the structure 1mbn.pdb. Here, only the four structures, 1gdj.pdb, 1jw8.pdb, 1lhs.pdb, and 1lht.pdb, are well matched to 1bmn.pdb. RMSD, root-mean-square deviation.

**Figure S2 Ten scatter plots of the feature points of 1bmn.pdb and the feature points of ten structures**

**Figure S3 Analysis of factors affecting the efficiency of FP-Zernike**

**A.** Correlation analysis between the number of feature points and the running time of FP-Zernike. **B.** The proportion of running time in PM-Zernike mode of FP-Zernike. Here, “ex-time” represents the time required to extract feature points from the structure representation, “re-time” represents the time required to generate the structure representation, “vox-time” represents the time required to voxelize the feature points, “geo-time” represents the time required to calculate geometric moments, and “g2z-time” represents the time required to compute the 3D Zernike descriptor from the geometric moments. **C.** The proportion of running time in PS-Zernike mode of FP-Zernike. **D.** Correlation analysis between the running time to generate the structure representation and the running time of FP-Zernike. **E.** The proportion of running time in GMM-Zernike mode of FP-Zernike. **F.** The proportion of running time in ATOM-Zernike mode of FP-Zernike.

**Figure S4 The relationship between the efficiency of FP-Zernike and the size of the structure**

**A.** In PM mode, the distribution of time taken to compute the descriptor of the structure based on FP-Zernike. Each color block in the panel implies the size of the structure. For example, 50–100 means that the structure contains 50–100 amino acids. For each size, we randomly selected 7–11 structures. **B.** In ATOM mode, the distribution of time taken to compute the descriptor of the structure based on FP-Zernike. **C.** In PS mode, the distribution of time taken to compute the descriptor of the structure based on FP-Zernike. **D.** In GMM mode, the distribution of time taken to compute the descriptor of the structure based on FP-Zernike.

**Figure S5 Demonstration of using our webserver**

**A.** Search service for protein chains. Users can input a protein database bank identity document (PDB ID) or upload a pdb file as a query structure to quickly find similar structures and view the 3D structure by clicking “view 3D”. **B.** Computational services on descriptors. Users can quickly get a structure descriptor. **C.** Descriptor computation service for multiple structures. Users can pack multiple structures into a zip archive and upload it to our webserver to receive all the descriptors of these structures in their provided e-mail. PDB, Protein Data Bank; ID, identity document.

**Tables**

**Table S1 Details of Protein160, Protein13, and RNA16**

**Table S2 Details of Protein160-Pairs, Protein13-Pairs, and RNA16-Pairs**

**Table S3 Comparative analysis of ReOmokage and Omokage (alignment of 31 protein structures with 1mbn.pdb)**

**Table S4 Performance evaluation of three metrics**

**Table S5 Efficiency analysis of four modes of FP-Zernike**
