# Supplementary material for: FP-Zernike: An Open-source Structural Database Construction Toolkit for Fast Structure Retrieval
Source: Genomics Proteomics Bioinformatics. 2024 Jan 19;22(1):qzae007. doi: 10.1093/gpbjnl/qzae007 (PMC11423855; doi:10.1093/gpbjnl/qzae007)
Supplement: qzae007_Supplementary_Data [file qzae007_supplementary_data.zip › TableS1-done.docx]

**Table S1 Details of Protein160, Protein13, and RNA16**

|  | **Protein160** | **Protein13** | **RNA16** |
| --- | --- | --- | --- |
| Structure type | protein | protein | RNA |
| Single chain | Yes | No | Yes |
| Number of structures | 1552 | 1431 | 955 |
| Number of groups | 160 | 13 | 16 |
| Class size distribution | 7–10 | 20–254 | 7–342 |
| Amino acid/base length distribution | 93–199 | 114–584 | 33–4010 |
