# Supplementary material for: FP-Zernike: An Open-source Structural Database Construction Toolkit for Fast Structure Retrieval
Source: Genomics Proteomics Bioinformatics. 2024 Jan 19;22(1):qzae007. doi: 10.1093/gpbjnl/qzae007 (PMC11423855; doi:10.1093/gpbjnl/qzae007)
Supplement: qzae007_Supplementary_Data [file qzae007_supplementary_data.zip › TableS2-done.docx]

**Table S2 Details of Protein160-Pairs, Protein13-Pairs, and RNA16-Pairs**

|  | **Protein160-Pairs** | **Protein13-Pairs** | **RNA16-Pairs** |
| --- | --- | --- | --- |
| Number of positive samples | 6722 | 130,671 | 83,839 |
| Number of negative samples | ${20,166}^{\times3}$ | ${392,013}^{\times3}$ | ${251,517}^{\times3}$ |

*Note*: Randomly select 3 times in the entire negative sample database, and select 20,166 structure pairs each time to form 3 data sets, denoted as “${20,166}^{\times3}$”. The meanings of “${392,013}^{\times3}$” and “${251,517}^{\times3}$” are the same as those of “${20,166}^{\times3}$”.
