# Supplementary material for: FP-Zernike: An Open-source Structural Database Construction Toolkit for Fast Structure Retrieval
Source: Genomics Proteomics Bioinformatics. 2024 Jan 19;22(1):qzae007. doi: 10.1093/gpbjnl/qzae007 (PMC11423855; doi:10.1093/gpbjnl/qzae007)
Supplement: qzae007_Supplementary_Data [file qzae007_supplementary_data.zip › TableS3-done.docx]

**Table S3 Comparative analysis of ReOmokage and Omokage (alignment of 31 protein structures with 1mbn.pdb)**

| **PDB ID** | **ReOmokage Score** | **Omokage Score** | **Difference** | **TM-score-TM-align** | **TM-score-DeepAlign** | **Shot** |
| --- | --- | --- | --- | --- | --- | --- |
| 1gdj | 0.03 | 0.86 | 0.83 | 0.53 | 0.00 | 0.00 |
| 1jw8 | 0.02 | 0.85 | 0.83 | 0.59 | 0.98 | 0.00 |
| 1gi5 | 0.25 | 0.82 | 0.57 | 0.13 | 0.00 | 1.00 |
| 1ss6 | 0.29 | 0.84 | 0.55 | 0.17 | 0.03 | 1.00 |
| 1v4r | 0.27 | 0.81 | 0.54 | 0.22 | 0.05 | 1.00 |
| 1lht | 0.48 | 0.88 | 0.41 | 0.97 | 0.65 | 0.00 |
| 1lhs | 0.48 | 0.87 | 0.39 | 0.96 | 0.66 | 0.00 |
| 1ywx | 0.45 | 0.80 | 0.35 | 0.20 | 0.00 | 1.00 |
| 3eb5 | 0.48 | 0.81 | 0.34 | 0.00 | 0.02 | 1.00 |
| 2oo2 | 0.50 | 0.82 | 0.32 | 0.22 | 0.00 | 1.00 |
| 1gdk | 0.59 | 0.88 | 0.29 | 0.53 | 0.00 | 1.00 |
| 3hon | 0.58 | 0.85 | 0.27 | 0.17 | 0.00 | 1.00 |
| 2kvo | 0.55 | 0.81 | 0.26 | 0.17 | 0.00 | 1.00 |
| 1v05 | 0.57 | 0.83 | 0.26 | 0.00 | 0.00 | 1.00 |
| 6h88 | 0.55 | 0.81 | 0.25 | 0.00 | 0.00 | 1.00 |
| 3bkf | 0.58 | 0.82 | 0.23 | 0.27 | 0.06 | 1.00 |
| 1pco | 0.61 | 0.82 | 0.21 | 0.16 | 0.00 | 1.00 |
| 4ndt | 0.61 | 0.82 | 0.21 | 0.23 | 0.00 | 1.00 |
| 2kb3 | 0.66 | 0.86 | 0.20 | 0.20 | 0.00 | 1.00 |
| 4npx | 0.64 | 0.83 | 0.19 | 0.22 | 0.05 | 1.00 |
| 2mga | 0.66 | 0.85 | 0.19 | 0.97 | 0.98 | 0.00 |
| 1aci | 0.62 | 0.81 | 0.19 | 0.20 | 0.04 | 1.00 |
| 1kva | 0.67 | 0.82 | 0.15 | 0.20 | 0.00 | 1.00 |
| 1xug | 0.66 | 0.81 | 0.15 | 0.13 | 0.00 | 1.00 |
| 1mbd | 0.76 | 0.89 | 0.13 | 0.99 | 0.99 | 0.00 |
| 1mbo | 0.78 | 0.90 | 0.12 | 0.99 | 0.99 | 0.00 |
| 1wrj | 0.69 | 0.80 | 0.12 | 0.21 | 0.00 | 1.00 |
| 2bk9 | 0.70 | 0.81 | 0.11 | 0.54 | 0.05 | 0.00 |
| 1ulp | 0.71 | 0.82 | 0.11 | 0.14 | 0.03 | 1.00 |
| 2mgb | 0.73 | 0.84 | 0.10 | 0.97 | 0.98 | 0.00 |
| 2dne | 0.73 | 0.84 | 0.10 | 0.16 | 0.00 | 1.00 |

*Note*: "TM-score-TM-align" is obtained by TM-align and "TM-score-DeepAlign" is obtained by Deepalign. "Shot = 1" indicates that ReOmokage score is more reasonable (closer to TM-score and pymol-RMSD).
