# Supplementary figures and images for: FP-Zernike: An Open-source Structural Database Construction Toolkit for Fast Structure Retrieval
Source: Genomics Proteomics Bioinformatics. 2024 Jan 19;22(1):qzae007. doi: 10.1093/gpbjnl/qzae007 (PMC11423855; doi:10.1093/gpbjnl/qzae007)

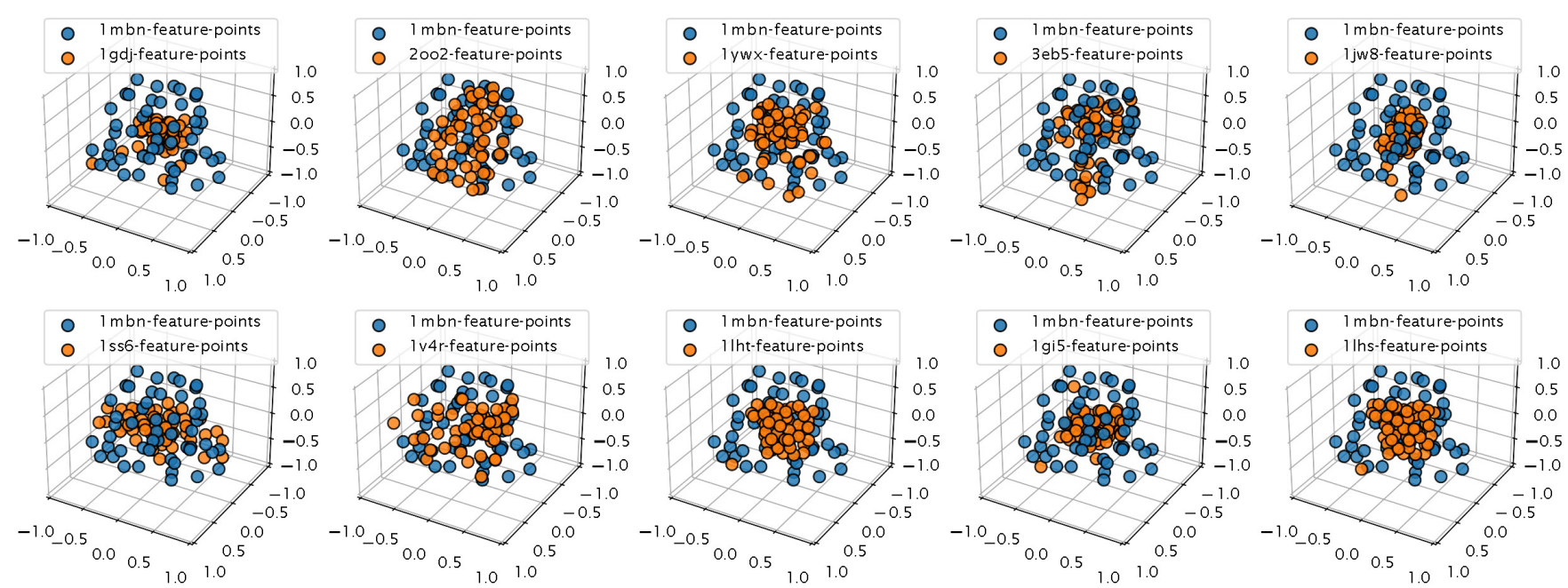

Supplement: qzae007_Supplementary_Data [file qzae007_supplementary_data.zip › Figure S2.pdf]

**A**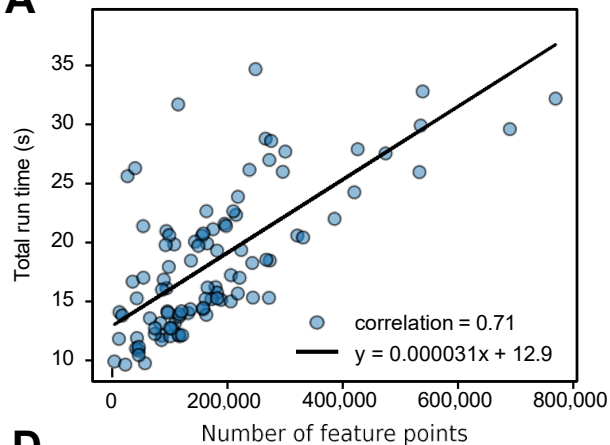**B**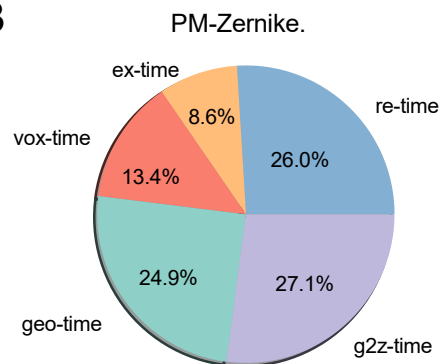**C**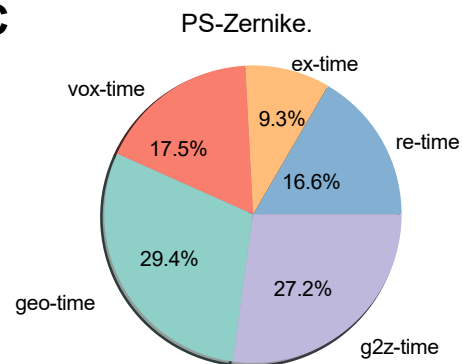**D**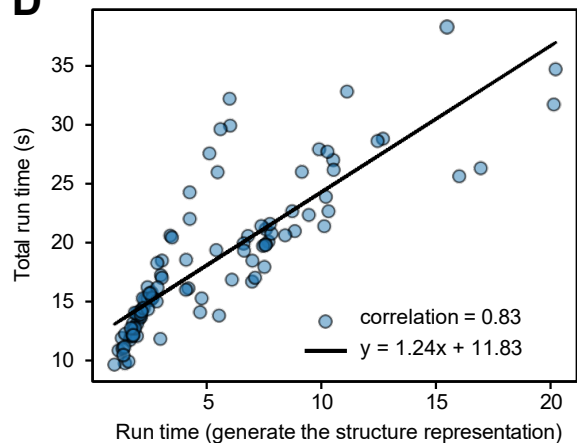**E**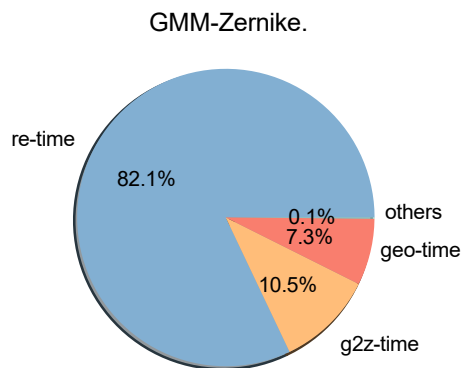**F**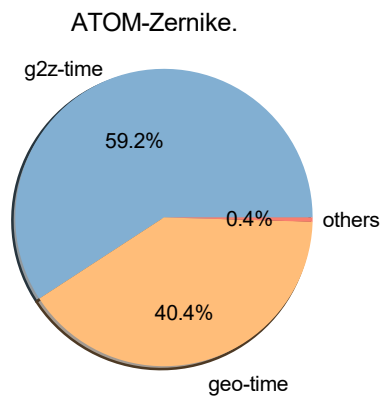

Supplement: qzae007_Supplementary_Data [file qzae007_supplementary_data.zip › Figure S3.pdf]
